# Mercury Pollution History in Tropical and Subtropical American Lakes: Multiple Impacts and the Possible Relationship with Climate Change

**DOI:** 10.1021/acs.est.2c09870

**Published:** 2023-02-21

**Authors:** Handong Yang, Laura Macario-González, Sergio Cohuo, Thomas J. Whitmore, Jorge Salgado, Liseth Peréz, Antje Schwalb, Neil L. Rose, Jonathan Holmes, Melanie A. Riedinger-Whitmore, Philipp Hoelzmann, Aaron O’Dea

**Affiliations:** † Environmental Change Research Centre, University College London, Gower Street, London WC1E 6BT, U.K.; ‡ Institut für Geosysteme und Bioindikation, Technische Universität Braunschweig, Langer Kamp 19c, D-38106 Braunschweig, Germany; § Tecnológico Nacional de México−I. T. de la Zona Maya, Carretera Chetumal-Escárcega Km 21.5, Ejido Juan Sarabia, 77965 Juan Sarabia, Quintana Roo, Mexico; ∥ Tecnológico Nacional de México−I. T. Chetumal, Av. Insurgentes 330, Chetumal 77013, Quintana Roo, Mexico; ⊥ University of South Florida, 140 7th Avenue South, St. Petersburg, Florida 33701, United States; # Programa de Ingeniería Civil, Grupo de Infraestructura y Desarrollo Sostenible, Universidad Católica de Colombia, Bogotá 111311, Colombia; ∇ School of Geography, University of Nottingham, Nottingham NG7 2RD, U.K.; ○ Smithsonian Tropical Research Institute, P.O. Box 0843-03092, Balboa 0843-03092, Panama; ◆ Institut für Geographische Wissenschaften, Freie Universität Berlin, Malteser Strasse 74-100, D-12249 Berlin, Germany

**Keywords:** lake sediments, secondary pollution, climate impact, human impact, atmospheric deposition, pollutants, tropics, subtropics

## Abstract

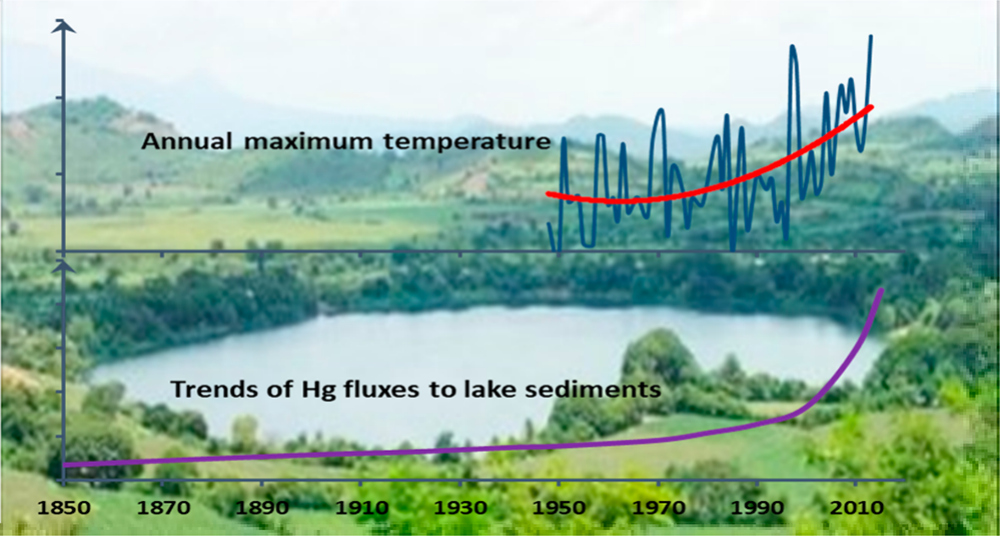

Sediment cores obtained from 11 tropical and subtropical American lakes revealed that local human activities significantly increased mercury (Hg) inputs and pollution levels. Remote lakes also have been contaminated by anthropogenic Hg through atmospheric depositions. Long-term sediment-core profiles revealed an approximately 3-fold increase in Hg fluxes to sediments from c. 1850 to 2000. Generalized additive models indicate that c. 3-fold increases in Hg fluxes also occurred since 2000 in the remote sites, while Hg emissions from anthropogenic sources have remained relatively stable. The tropical and subtropical Americas are vulnerable to extreme weather events. Air temperatures in this region have shown a marked increase since the 1990s, and extreme weather events arising from climate change have increased. When comparing Hg fluxes to recent (1950–2016) climatic changes, results show marked increases in Hg fluxes to sediments during dry periods. The Standardized Precipitation–Evapotranspiration Index (SPEI) time series indicate a tendency toward more extreme drier conditions across the study region since the mid-1990s, suggesting that instabilities in catchment surfaces caused by climate change are responsible for the elevated Hg flux rates. Drier conditions since c. 2000 appear to be promoting Hg fluxes from catchments to lakes, a process that will likely be exacerbated under future climate-change scenarios.

## Abstract 2

The sediment records from 11 tropical and subtropical American lakes show marked increases in mercury inputs to the lakes since c. 2000, caused by local human and natural impacts and likely related to recent climate change.

## 1 Introduction

Mercury is a global pollutant of concern because of the potential for dietary exposure to methylmercury (CH_3_Hg^+^). Mercury is ranked third among the most toxic elements for human health according to the Agency for Toxic Substances and Disease Registry (ATSDR) in the United States.^1^ It is estimated that there is a health risk associated with mercury exposure for 19 million people worldwide, with a possible disease burden of 1.5 million disability-adjusted life years.^2^ Mercury emissions to the atmosphere from human activities have undergone substantial increases globally through the industrial period, and present-day Hg atmospheric emissions from primary anthropogenic sources are estimated to be about 2000 Mg year^–1^. By comparison, natural geogenic emissions are estimated to be approximately 500 Mg year^–1^.^3,4^

Lakes play an important role in the biogeochemical cycle of Hg. Inorganic Hg deposition from lake catchments can be converted to toxic methylmercury or re-emitted to the atmosphere.^5^ Mercury also can be captured in lake sediments through geochemical and biological processes. Sediment cores serve as natural historical archives and have been used widely to assess past environmental changes, especially when little or no long-term monitoring data are available.^6^ Consequently, lake sediment studies provide context for modern observations and are useful for uncovering drivers of change. Based on a compilation of historical archives, principally lake sediment cores, it is estimated that Hg in atmospheric deposition to remote aquatic ecosystems has increased around 3- to 5-fold globally since the 1850s.^4,7−10^

In remote lakes, as no anthropogenic Hg sources exist locally, anthropogenic Hg is transported to the site only through atmospheric deposition. Therefore, the sediment record from a remote lake may show the atmospheric pollution history of the area. However, if a lake has been directly affected by human activities, such as mining, or land use change in its catchment, these activities may disturb catchment soil stability, resulting in elevated Hg transport from the catchment to the lake or bringing new Hg source to the lake. As a consequence, the sediment Hg record from a lake directly affected by human activity may not reflect the true history of atmospheric Hg deposition. Close links between climate change and soil erosion have been observed in the past decades,^11^ and soil erosion is expected to have increased in many locations worldwide.^11,12^ With climate change becoming more severe in many areas, it has become an important factor affecting soil stability. Hence, climate change could potentially cause more Hg stored in catchment soils to be eroded and transported into lakes, even in the remote sites, resulting in elevated Hg inputs to the sediments.

Mercury-pollution research based on studies of lake sediments has been undertaken intensively in many regions of the world. However, current and historical trends of Hg pollution in lakes of the tropical and subtropical Americas are spatially and temporally sparse. In addition to anthropogenic influences, this region is of particular interest because it has been described as highly vulnerable to climate change.^13^ Honduras, for example, is often listed as the most vulnerable country in the world, as indicated by the Global Climate Risk Index 2018, and is the country that was most affected by climate change from 1996 to 2016.^14^ Changes in temperature and precipitation regimes across the tropics and subtropics in the Americas have resulted in a greater frequency of extreme climate events such as droughts and floods, and the severity of these is expected to increase over the coming decades.^15^ Meanwhile, it is widely acknowledged that we are in a changing world, but the processes by which climate change has affected inputs of pollutants into lakes both now and in the future are not well understood. Because sediment records allow a comparison of undocumented periods in the past with modern conditions, they provide the means to assess the scale, extent, and rate of changes in the Hg presence in the environment.

In this study, long-term changes in Hg content were examined in radiometrically dated (1850–2017) sediment cores collected from tropical and subtropical American lakes. The lakes were selected to represent gradients in human impact and climate with the aim of better understanding the broader dynamics of Hg deposition in lakes of this region. Sediments were analyzed for Hg content to assess spatial and temporal Hg pollution patterns. Importantly, since the region is vulnerable to climate change, by looking at sediment Hg inputs in the remote lakes where direct human impacts can be excluded, this study addresses the potential impact of climate change on Hg content in sediments.

## 2 Methods

### 2.1 Study Sites

**Figure 1:**
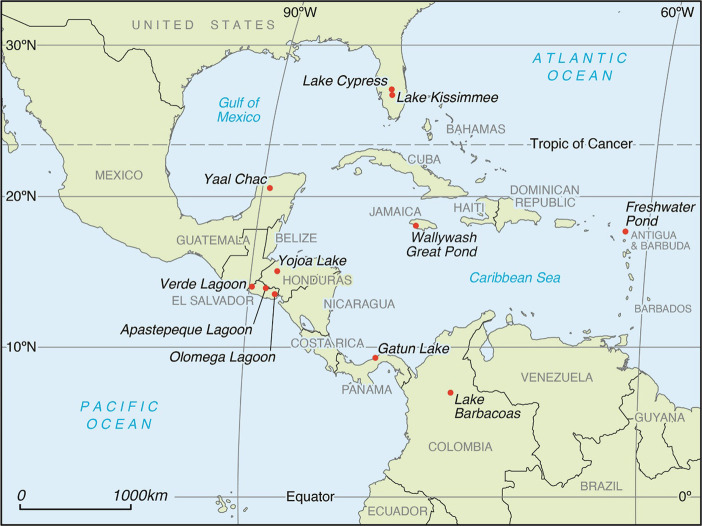
Distribution of study lakes in tropical and subtropical Americas.

The region of study spans the northern tropical and subtropical zones of the Americas (Figure 1). The region is geomorphologically and geographically variable^16^ and contains karst, tectonic, and volcanic lakes, all of which are represented in this study. Sediment cores were collected in 11 natural and man-made lakes in southern North America (Florida, USA), the Caribbean (Jamaica, Barbuda), Central America (Panama, Honduras, El Salvador, and Mexico), and the northern portion of South America (Colombia). Table 1 presents information about specific locations, catchment and lake areas, and codes that are used to refer to sediment cores in the text.

**Table 1:** Information of Study Lakes and Coring Times in Tropical and Subtropical Americas^a^

country	lake	code	latitude	longitude	lake area (km^2^)	catchment area (km^2^)	coring year
USA (Florida)	Lake Cypress	CY40	28°04′40.3″N	81°19′02.8″W	16.6	93.2	2017
USA (Florida)	Lake Kissimmee	KIS43	27°53′49.8″N	81°15′38.5″W	143.7	535.3	2017
Mexico	Yaal Chac	YCH	20°35′43.065″N	89°42′40.468″W	0.01	0.013	2013
Jamaica	Wallywash Great Pond	WAGP1	17°58′17.8″N	77°48′25.4″W	0.38	1.19	2013
Barbuda	Freshwater Pond	BFWP	17°36′05.8″N	61°47′45.2″W	0.17	3.61	2010
Honduras	Yojoa Lake	YOJOA	14°49′34.57″N	87°59′29.85″W	78.8	764	2013
El Salvador	Verde Lagoon	VERDE	13°53′29.28″N	89°47′13.83″W	0.64	1.1	2013
El Salvador	Apastepeque Lagoon	APAS	13°41′32.84″N	88°44′42.41″W	0.37	11.78	2013
El Salvador	Olomega Lagoon	OLOM	13°18′26.04″N	88°03′18.27″W	23.3	79.7	2013
Panama	Gatun Lake	LGAT1	9°2′49.58″N	79°50′6.33″W	344	879^b^	2013
Colombia	Lake Barbacoas	LBARB1	6°44′26″N	74°14′36″W	9.0	233.5	2016

a The catchment area does not include the lake area.

b Catchment area in Gatun Lake was calculated when the lake area is 344 km^2^.

Lakes Cypress and Kissimmee are located in the Kissimmee Basin of south Florida, USA. These lakes originally held “pure, clear water”,^17^ but after being connected by engineered canals in 1884, water quality was degraded.^18^ Human settlement increased in the Kissimmee Basin after the American Civil War (c. 1865), and the region was subsequently developed extensively for agriculture and cattle ranching. After World War II, urban development and point-source nutrient discharges affected the lakes and water-quality deterioration became increasingly evident. Discharges from the lakes currently affect downstream water quality in Lake Okeechobee and the Florida Everglades, the largest Ramsar Wetland of International Importance in the USA.^19^

Lake Yojoa is one of the few freshwater lakes in Honduras. It lies in a depression formed by volcanic activity with a relatively large surface area (Table 1). The lake catchment is under the protection of Santa Bárbara National Park and Cerro Azul Meámbar National Park. The Lenca Culture developed here and persisted for centuries, and there are currently few towns within the catchment. A highway passes the catchment and connects the country’s two largest cities. A zinc-lead-silver mine operated since 1938 in El Mochito, about 6 km to the west of the lake, from which wastewater has been discharged into streams that flow from the northwest toward the lake.^20,21^

Apastepeque Lagoon is a maar lake in Salvador, and it is fed by direct precipitation. Part of the catchment around the lake has been managed for cultivated crops in recent years, and lake water quality has degraded. The lagoon is a popular recreational site, with restaurants and a tourist center along its shores.

Gatun Lake in Panama is one of the largest and oldest artificial lakes in the world. The lake was built in 1913 to form what today is the Panama Canal. The lake serves as a channel to facilitate global trade and cross-oceanic travel and as a freshwater reservoir that is used to generate hydropower that supplies Panama City and other surrounding towns.^22,23^ A long monsoon season with high rainfall and a short dry season dictate the hydrodynamics of the lake.^23^

Lake Barbacoas is an oxbow floodplain lake located on the western side of the Magdalena River in Colombia. The catchment of Magdalena River has been heavily altered over the past seven decades in response to expanding agricultural, industrial, and urban frontiers, which together have led to an increase in pollutant and sediment load into the river.^24,25^ Illegal gravel and gold-mining activities also have been important emergent stressors,^26^ along with increased deforestation rates since the late 1990s.^24^ Because of its connectivity to the Magdalena River, it has been proposed that the lake is hydrologically more dependent on ENSO events than on local human controlling factors.^27^

The remaining five lakes in this study are undisturbed lakes located in rural areas of low population density or in remote areas, so they may be used to assess atmospheric Hg deposition and possible climate impacts. Yaal Chac is a karst lake in Mexico, and Verde Lagoon is a crater lake in El Salvador. Wallywash Great Pond in Jamaica is a marl lake that is situated in a fault-bounded basin in the Oligo-Miocene Newport Formation of the White Limestone Group in the Parish of St. Elizabeth. All three of these lakes are fed by direct precipitation.^28^ Freshwater Pond in Barbuda is a permanent inland freshwater to brackish-water lake that is closed hydrologically and is situated on the Codrington Limestone Group. Olomega Lagoon, the largest freshwater lake in the southeastern part of El Salvador, is located in the Central American Dry Forest Ecoregion in an area of low population density without any perceivable industrial activity in the catchment. It is a Ramsar site that is subject to control for human impact.

### 2.2 Core Collection and Radiometric Dating

Sediment cores were taken with gravity and push-rod corers from shallow areas of Gatun and Barbacoas and from deep areas of the other lakes between 2010 and 2017. The various projects that contributed to the present study generally included the analysis of multiple biological and geochemical indicators in the lakes. Most sediment cores were sliced in 0.5 or 1 cm intervals throughout their total lengths, whereas cores CY40 and KIS43 from Florida lakes were sliced in 5 cm intervals for the top 20 cm and then 4 cm intervals through the rest of the cores because of very rapid (∼1 cm/year) sedimentation rates. Sediments were measured for bulk dry densities and freeze-dried.

All sediment cores were radiometrically analyzed for ^210^Pb, ^226^Ra, ^137^Cs, and ^241^Am by direct gamma assay at the UCL Environmental Radiometric Facility and were dated using the constant rate of the ^210^Pb supply (CRS) dating model.^29^ Dry mass sediment accumulation rates through time were derived from these chronologies. To estimate sediment and Hg accumulation rates beyond the limits of ^210^Pb dating, we extrapolated mean sediment accumulation rates of the mid- to late 19th century to the base of cores. Extrapolations exert limited adverse influence on reconstructed Hg pollution estimates because the time frame for the pollution that we address occurred mainly in the past 100 to 200 years.

### 2.3 Mercury Analysis

Total Hg was analyzed using cold vapor-atomic fluorescence spectrometry (CV-AFS) following reduction with SnCl_2_. Freeze-dried samples were digested with 8 mL of aqua regia at 100 °C on a hotplate for 2 h in rigorously acid-leached 50 mL polypropylene digestion tubes. Standard reference material that consists of stream sediment (GBW07305; the certified Hg value is 100 ng g^–1^) and sample blanks were digested with every 20 samples or less. Average reference material recoveries were within 4% of certified values, with standard deviation (SD) less than 5 ng g^–1^ (*n* > 3) for every batch of sample digestions and measurements. During the CV-AFS measurements, standard solutions and quality-control blanks were measured every five samples to monitor measurement stability.

### 2.4 Data Analyses

Generalized additive models (GAMs, “mgcv” package in R, https://cran.utstat.utoronto.ca/web/packages/mgcv/mgcv.pdf) were used to estimate significant trends of temporal changes in Hg accumulation rates using smooth functions. GAMs were fitted to ratios calculated as Hg accumulation rates at points in time divided by pre-1850 rates,^30^ and the residual maximum-likelihood (REML) method was used to penalize overfitting trends. A Gaussian distribution with an identity link was used to model the time-series data, and diagnostic Q–Q plots were performed to check for homogeneity of variances in the residuals. A base function (*k*) of 15 was used to achieve the best model fit. The first derivative function of each GAM was also identified and used to determine significant trends in the time-series data using the “gratia” package in R (https://gavinsimpson.github.io/gratia/). Here, trends that deviated from 0 (no trend) indicated periods of significance.^31^ Two sets of GAMs were determined for (i) the dataset containing all lakes and (ii) the dataset containing only undisturbed lakes.

To quantify how Hg ratios in the study lakes relate to extreme long-term climatic events (El Niño–Southern Oscillation (ENSO)), we ran a Standardized Precipitation–Evapotranspiration Index (SPEI) analysis.^32^ This analysis uses historical climate data to generate a drought index based on the difference between precipitation and potential evapotranspiration rates across a given area, which allows identification of years with extreme drought or excess precipitation.^32^ The SPEI data were downloaded from https://spei.csic.es/map/maps.html#months=1#month=3#year=2020 for the interval between 1950 and 2016. Years with severe drought or excess precipitation were obtained using annual mean data (i.e., 12 month timescale). The Global SPEI database is based on worldwide monthly drought condition data with a spatial resolution of 0.5°. Years with SPEI^32^ between −0.5 and 0.5 are considered to fall within normal conditions. Years with values >2 are considered to be extremely wet, and values <−2 are considered as extremely dry.^32^

To address more specifically the extent to which SPEI data relate to sediment Hg flexes, we used Boosted Regression Tree analysis (BRT; ref (33)). BRT was used to partition the variation in Hg flexes explained by SPEI and land-use-change descriptors alone. BRT constitutes a machine-learning method that combines classical regression-tree analysis with boosting.^33^ BRT is appropriate for this study because it can accommodate collinear data and handle nonlinear descriptors with missing values. BRT partitioning (pBRT) was assessed through an additive partial regression scheme following Feld et al.^34^ This analysis decomposed each BRT-explained variation into four fractions: (i) pure climate (SPEI), (ii) pure land use, (iii) shared climate/land use, and (iv) unexplained variation. The shared fraction (iii) represents the variation that may be attributed to climatic and/or land-use descriptors together, and it is obtained additively by partial regression. Land-use descriptors were derived from the annual (2000–2016) and decadal (1950–2000) variation in total estimated areas of pastures (meadows and grasslands) in each of the study lake countries presented in the Hyde 3.2 database^35^ for the period 1950–2016. The pBRT was run using Gaussian distributions with a tree complexity of 2, a learning rate of 0.01, and a bag fraction of 0.5. The set.seed (123) argument in R was used to seed the BRT as a numerical starting point under the “dismo”^36^ and “gbm”^37^ packages in R.^38^

## 3 Results

### 3.1 Sediment Chronologies and Accumulation Rates

Sediment chronologies and accumulation rates of the cores are provided in the Supporting Information. All the sediment cores extended back to a period prior to the 1850s, except for the LBARB1 core (Lake Barbacoas, Colombia), which spanned only the past six decades (1960–2016, Figures S1–S22). In most cores, bulk sediment accumulation rates were relatively low, generally <0.03 g cm^–2^ year^–1^ before 1960, with the exception of the Cypress Lake (Florida) core (CY40), in which rates reached 0.14 g cm^–2^ year^–1^ in the 1920s. After c. 1960, sediment accumulation rates progressively increased in most of the cores, especially in LBARB1 (Colombia), LGAT1 (Panama), and BFWP (Barbuda) (Figure S23). The LBARB1 core showed the highest bulk sedimentation rates among all lakes, with a value of 1.4 g cm^–2^ year^–1^ in 2016 (Figure S4).

### 3.2 Hg Concentrations and Fluxes

**Figure 2:**
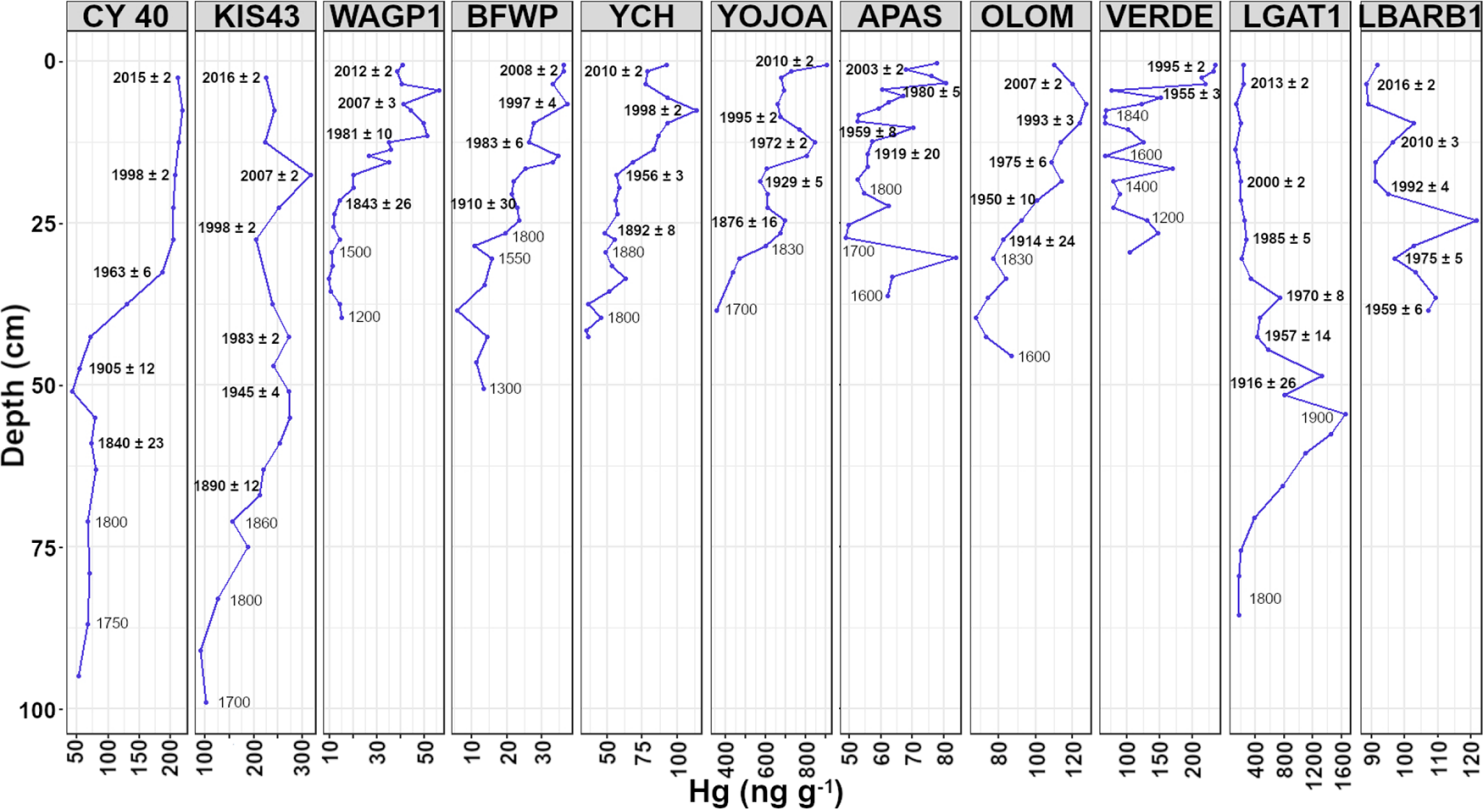
Mercury concentrations versus depth in the sediment cores. The years (AD) marked with the bold font are derived from the ^210^Pb CRS model, while the years marked with the light font are estimated based on the ^210^Pb sedimentation rates.

In most cores, Hg concentrations in the pre-1800 sediments were generally low, ranging from 10 to 50 ng g^–1^. A few anomalous Hg concentration peaks were recorded in the older (pre-1850s) sediments of some lakes, including a peak around c. 1500s in the core from Verde Lagoon (VERDE) and a peak around the mid-1600s in Apastepeque Lagoon (APAS) (Figure 2). Verde Lagoon and Apastepeque formed in volcanic craters, and the areas where these lakes are located were subject to several eruptions from local volcanoes during the 16th and 17th centuries.^39^ Older sediments at these sites, therefore, apparently were influenced by volcanic Hg.

The rates of increase in Hg concentrations in the sediment cores were not uniform through time. In general, there was a gradual increase from c. 1850 to recent decades (Figure 2). A general pattern of increasing Hg concentration is consistent with the rise of anthropogenic Hg observed in many other archives worldwide.^4,7,8,40,41^ Given the shorter time span of the LBARB1 core (Barbacoas Lake), the early phase (pre-1950s) of Hg increases was not well represented.

**Figure 3:**
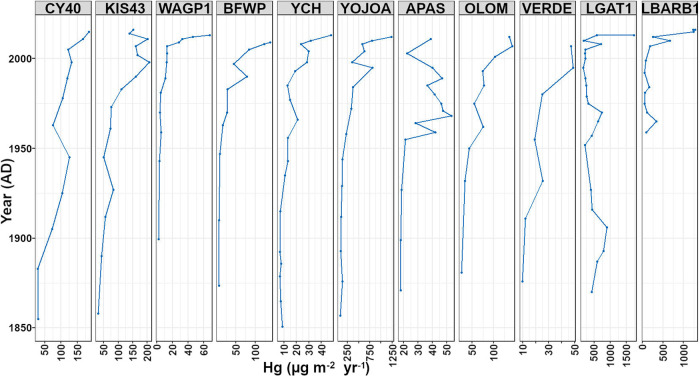
Mercury fluxes versus time in the sediment cores.

**Figure 4:**
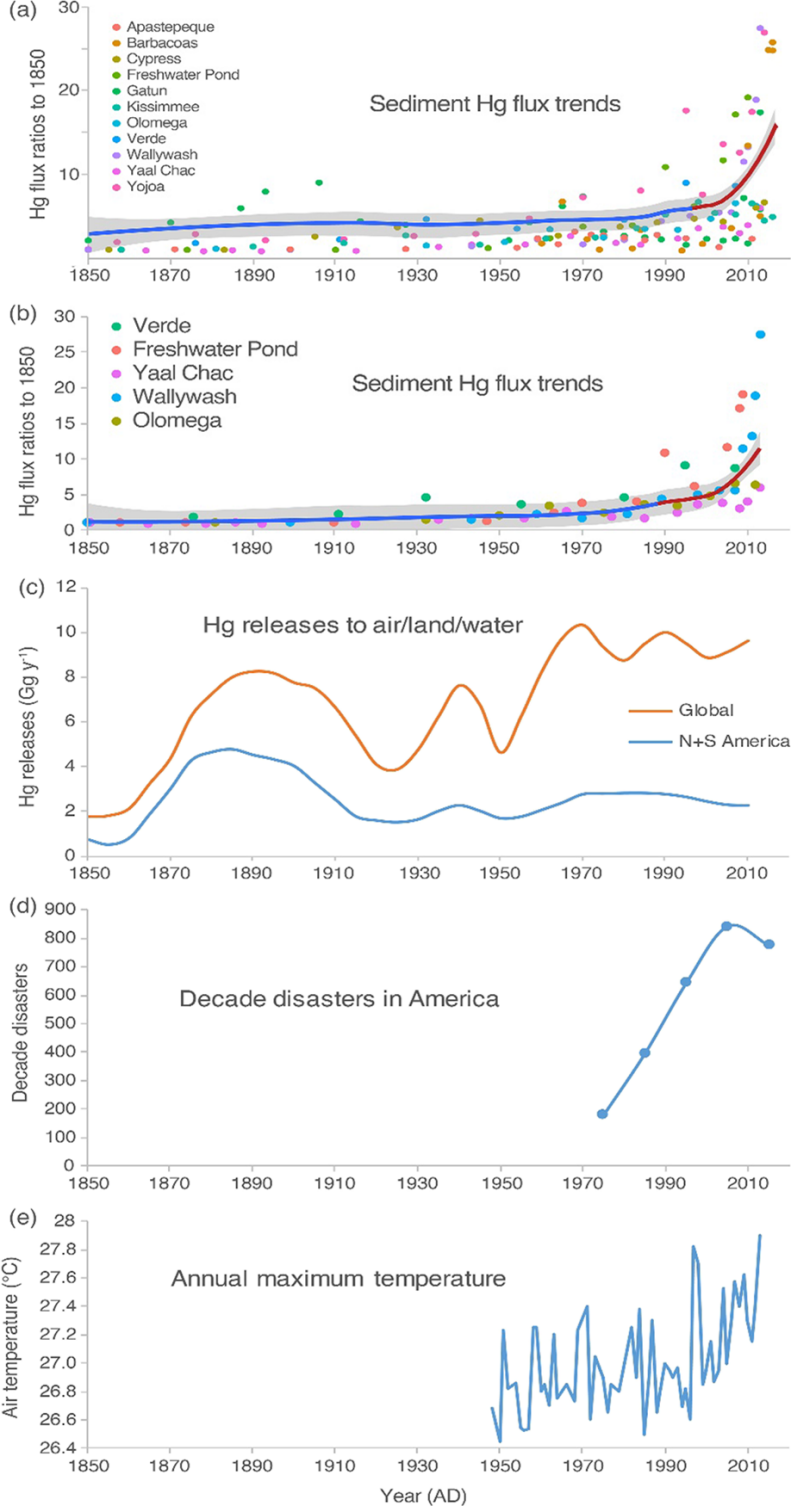
Possible impacts on increased Hg inputs into the lakes in tropical and subtropical American region since 2000. (a) Hg flux ratio trends derived from the GAMs in the sediments in all the study lakes (ratios are against the 1850 fluxes, except Yojoa (the 1700 flux), Gatun (the 1800 flux), as local human activities occurred in both lakes around the 1850s, and Barbacoas (the 1990 flux, the lowest in the core), as the core covers the 1960s to 2016); (b) Hg flux ratio trends in the lakes where anthropogenic Hg solely originates from atmospheric deposition; (c) global Hg release to the environment from anthropogenic sources and Hg release to the environment from anthropogenic sources in America;^57^ (d) reported average decade disasters in South America, North America, Central America, and the Caribbean;^78^ (e) annual maximum temperature in the Intra-Americas Region.^68^

Mercury fluxes to the sediments generally show a three-phase pattern with a gradual increase from 1850 to around the 1950s, an intensification from the mid-1990s to the early 2000s, and a third phase in which Hg depositional rates increased more rapidly since c. 2000 (Figure 3). Statistical results for the generalized additive models revealed a significant threshold of increase in Hg deposition among the lakes after the mid-1990s, and from this point, Hg fluxes gradually increased nearly 4-fold to the present time (Figure 4a). An increase in Hg deposition patterns after the mid-1990s was evident even in the least undisturbed set of lakes, with Hg fluxes increasing c. 3-fold against the mid-1990s level (Figure 4b).

Table 2 shows basic features of Hg flux changes during different time periods. The 1850 fluxes reflect background levels in most of the cores. High fluxes in cores LGAT1 and YOJOA, however, reflected high Hg concentrations (200 and 640 ng g^–1^, respectively) in sediments formed at that time. Increases in Hg fluxes became more rapid after the 1950s in many of the cores, and by 1990, fluxes increased 2- to 5-fold compared with the 1850 levels.

**Table 2:** Mercury Fluxes (μg m^–2^ year^–1^) in the Sediment Cores at Different Times and Their Ratios against the 1850 Levels^a^

	core	Hg flux		
lake	time (AD)	1850	1990 (ratio to 1850)	2010 (ratio to 1850)
Lake Cypress	CY40	28	119 (4.2)	158 (5.6)
Lake Kissimmee	KIS43	31	160 (5.1)	180 (5.8)
Yaal Chac	YCH	6.5	18 (2.8)	31 (4.7)
Wallywash Great Pond	WAGP1	2.5	10.8 (4.3)	30 (12)
Freshwater Pond	BFWP	7	78 (10.8)	138 (19.1)
Yojoa Lake	YOJOA	87 (1.9 ratio to the 1700s)	400 (8.7, ratio to the 1700s)	800 (17.3, ratio to the 1700s)
Verde Lagoon	VERDE	7	35 (5)	47 (6.7)
Apastepeque Lagoon	APAS	17	46 (2.7)	30 (1.7)
Olomega Lagoon	OLOM	24	76 (3.2)	140 (5.8)
Gatun Lake	LGAT1	211(2.1, ratio to the 1800s)	319 (3.2, ratio to the 1800s)	800 (8, ratio to the 1800s)
Lake Barbacoas	LBARB1	87 (1960)	45	669 (>14.8)

a Since Hg concentrations have increased by local anthropogenic sources in the YOJOA and LGST1 cores in 1850, sediment background fluxes were calculated using the Hg concentrations in the earlier sediments at the bases of the relevant cores.

### 3.3 Climate and Land Use

SPEI analysis showed that except for Gatun and Barbacoas lakes, there was a general tendency for a gradual change from historical (1960–1990) wet conditions toward drier conditions after the 1990s (Figure S24). For Gatun and Barbacoas lakes, this long-term climatic pattern was much weaker, but both lakes experienced more extreme wet and dry periods after the 1990s as compared with conditions in their earlier histories. Mercury flux ratios generally declined under wetter conditions and increased under drier conditions (Figure S25).

Overall, land use was positively correlated with Hg fluxes among the lakes, and there was a tendency for more intensive land use in recent times. Lakes Cypress, Kissimmee, and Wallywash were exceptions because of their longer periods of human influence. BRT analysis revealed that climate alone explained 24% of the Hg long-term variation, while land use alone explained 38% of Hg variation. Another 31% of Hg long-term variation was explained by the shared component of climate/land use, while the remaining 7% of variation was unexplained.

## 4 Discussion

### 4.1 Multiple Impacts on the Lakes

#### 4.1.1 Direct Human Impacts in the Catchments

Some study sites revealed evidence of direct Hg contamination. For example, cores taken from the two Florida lakes, which are connected to upstream lakes that received wastewater discharge in past decades, showed generally higher Hg concentrations in modern sediments compared with records from the more remote lakes (e.g., Wallywash, Freshwater Pond, and Yaal Chac). Recent increases in Hg concentrations in the Florida lakes might have been affected by fossil-fuel consumption associated with large-scale engineered drawdowns and machine scraping of nearby lakes to remove organic matter accumulations.^18^

The core from Yojoa Lake, Honduras, reveals high Hg concentrations throughout. Concentrations in cores from neighboring countries (e.g., El Salvador and Jamaica) were generally less than 75 ng g^–1^, whereas concentrations even in the basal sediments of the Yojoa core are 355 ng g^–1^. There are two apparent sources for the high Hg levels in Yojoa: volcanic leaching and mining. Lake Yojoa was formed by volcanic processes. The nearby Ilopango caldera erupted several times during the Holocene and more recently between AD270 and AD535.^42^ Post-eruption leaching of Hg often has immediate effects on Hg concentrations in sediments, but concentrations typically decline with time. Leaching of volcanic material could explain some of the previous high levels of Hg in Yojoa, but it cannot explain the increase that was observed to the present day. Gold and silver mining is a known cause of Hg pollution in the region in the recent past and may be increasing,^43,44^ while local historical mining has stretched back centuries associated with development of Lenca cultural activities.^45,46^ A peak in Hg concentrations in the 1970s (Figure S26) coincides with peaks in Cd, Pb, and Zn, all of which are primarily derived from mining activities^20^ but cannot be attributed to a recent volcanic event. Volcanic processes therefore account for a portion of high Hg observed in early sediments of Yojoa Lake, but recent metal deposition is best explained by the establishment and modern intensification of nearby mining activities.

The core from Gatun Lake (LGAT1), Panama, shows an extremely high peak in Hg concentration of 1650 ng g^–1^ around the late 1800s to the early 1900s. Spheroidal carbonaceous particles (SCPs) are abundant in sediments of the same age and are products of high-temperature fossil-fuel combustion. This suggests that high Hg concentrations in c. 1900 also were derived from fossil fuels. The peaks coincide with the construction of the Panama Canal when coal was used widely to power construction machinery and with other human activities around the lake during the building of the dam and the Panama Canal.^23^

The relatively constant but high Hg concentrations in Barbacoas Lake (LBARB1 core), Colombia, suggest six decades of contamination. However, the lake sediments did not entirely reflect the development and magnitude of industrial and urbanization pollution in the Magdalena River basin since the 1950s.^47^ Barbacoas Lake is influenced largely by ENSO, which might have obscured the pollution signal of local factors in lake sediments.^27^ A marked increase in bulk sedimentation (around 10 times the baseline value; Figure S4) since the early 2000s suggests regional deforestation and climate effects^24,27^ that led to erosion that diluted Hg concentrations in sediments. However, erosion caused by catchment deforestation has brought a massive increase in Hg inputs to the lake, as shown by increased Hg fluxes into the sediments. Another lake (Lake Antoine, Grenada) in this region also shows a great increase in sediment Hg fluxes caused by catchment land use.^48^ Currently, methylmercury values in the lower part of the Magdalena River (north from Barbacoas Lake) are three to four times higher than natural background values.^49−51^

#### 4.1.2 Atmospheric Hg Deposition in the 19th and 20th Centuries

Hg records in sediment cores from lakes that experience little local direct impact more accurately reflect the dynamics of anthropogenic Hg that arises from atmospheric Hg deposition. In our study, these lakes include Verde Lagoon, Yaal Chac, Freshwater Pond, and Wallywash Pond. Mercury concentrations in recent sediments from these lakes are low (mostly in 40–100 ng g^–1^, Figure S27) compared with lakes that receive direct human impacts, but each of these lakes shows increasing Hg concentrations (Figure 2). Verde Lagoon is a crater lake, and recent increases in Hg concentrations to a relatively high level may be caused by very slow sediment accumulation that enriched atmospherically deposited Hg.^52^

Global atmospheric Hg emissions from coal combustion, a major source of atmospheric contaminants in modern history, have shown a gradual increase from the 1850s to the present.^53^ Artisanal and Small-Scale Gold Mining (ASGM) has also become a major source for Hg emission, at least in this region,^44^ and ASGM Hg emission from this region is likely to have also increased in recent years.^54^ Atmospheric Hg deposition arrives to lakes in two ways. The first is by direct atmospheric deposition, and the second is Hg that was deposited onto the terrestrial catchments and later reached lakes via runoff. If the terrestrial transported fraction is constant, then sediment Hg records can be used to assess trends in atmospheric Hg deposition.^55,56^

The Hg profiles of the Yaal Chac core (YCH), Freshwater Pond core (BFWP), and Wallywash Great Pond core (WAGP1) and those from the Apastepeque (APAS) and Verde (VERDE) crater lakes all show an approximately 2-fold increase in Hg concentrations in the past hundred years or so (Figure 2). Half of the study lakes are likely to have received Hg solely from the atmosphere, yet increases in Hg deposition were a common feature. If there were volcanic influences, the Hg profiles would have changed at the points where the influences occurred. The similarity of these patterns at diverse remote sites suggests that volcanic and direct human influences are not the sources of recent increases in Hg concentrations.

Mercury fluxes increased in the majority of the studied cores from the 1850s to the 1950s. In the directly affected lakes, local human activities might have made a contribution to this increase. However, in the undisturbed lakes, the Hg increase should be derived from increased atmospheric Hg deposition, if there has not been any increased catchment erosion. Increased Hg fluxes during this time frame have been documented in sedimentary records from remote lakes in other regions worldwide.^7,8,40,41,48^ A common view of the global Hg cycle is that there has been a 2- to 5-fold increase in atmospheric Hg deposition to remote areas since 1850 that resulted from increases in Hg emissions to the atmosphere from anthropogenic sources.^4,45^ Biogeochemical modeling showed a 3.2-fold increase in the atmospheric Hg burden relative to 1850,^57^ which is in line with previous findings. In a similar manner, our study showed that Hg fluxes increased 2- to 5-fold in most of our lakes from 1850 to 1990 (Table 2). Mercury flux rates in our undisturbed lake cores show an overall 3-fold increase in Hg fluxes by the mid-1990s as compared with 1850.

### 4.2 Rapid Increase in Hg Fluxes since 2000

To consider atmospheric Hg deposition that does not include local direct Hg inputs to the lakes during GAM analysis, we excluded cores YOJOA, LGAT1, and LBARB1 as they were affected by local human direct impacts from mining, construction, and deforestation, cores CY40 and KIS43 that apparently were affected by human activities in the catchments, and the APAS core because of recent changes in managed cultivation in its catchment.

Our analysis has shown for the first time that, in the lakes that have not been directly affected by human activities in the catchments, a new pollution pattern has emerged since c. 2000. By the early 2000s, overall fluxes increased nearly 4-fold, but by 2010, overall increases were approximately 9-fold in these lakes, a remarkable increase in flux rates (Figure 4b).

Since the 1970s, global total Hg release to all environments by human activities has been relatively stable around 9.5 Gg year^–1^, while anthropogenic Hg emission to the atmosphere has been stable at around 2 Gg year^–1^. In South and North America, the total Hg release of anthropogenic Hg emissions to the atmosphere declined slightly from the 1970s to 2010 (Figure 4c).^57^ From 2010 to 2015, there was an approximately 20% increase in global emissions of Hg to the atmosphere from anthropogenic sources, reaching a total of 2.22 Gg year^–1^.^58^ However, such anthropogenic emissions account for about 30% of Hg emitted annually to the atmosphere. A further 60% of current global Hg emissions to the atmosphere results from environmental processes, many of which involve recycling of anthropogenic Hg previously deposited to soils and water, while the final 10% is from natural sources such as volcanic emissions.^58^ This suggests that increased Hg emissions to the atmosphere from direct anthropogenic sources from 2010 to 2015 only account for about 6% of atmosphere Hg, which is far less than the increased Hg fluxes to sediments during that time period in the present study.

If we examine Hg releases from anthropogenic sources in the Americas more closely, they each have their own stories.^4,59^ Sources in North America reduced Hg release to the atmosphere from 1970 to 2010, while those in South America increased during the same period. However, the increased rate of Hg release in South America slowed after c. 2000.^45^ Hence, the pace of Hg release in South America does not fit the changes in recent Hg fluxes to sediments that is shown by the present study (also see the Supporting Information, Section 12). These facts indicate that changes in anthropogenic Hg emission to the atmosphere are not likely to be the primary reason for the observed increases in Hg fluxes to the lake sediments after c. 2000.

Although volcanic eruptions can affect Hg deposition in lakes, there has not been a general increase in global volcanic activity in recent years.^60^ Compiled volcanic emission data (Table S1) similarly indicate that there has not been an increase in volcanic emissions in the study region (Figure S29).

Large amounts of Hg emitted into the atmosphere as a global pollutant have been transported to remote areas and deposited in lakes and their catchments,^61−63^ and as a result, a lake’s catchment can be a large Hg storage pool^64^ such that catchment inputs are important sources for Hg transported to lake sediments.^65^ If direct atmospheric deposition of Hg has not increased sufficiently to account for the observed trends in our lakes that have limited local human activities, then the increases in Hg fluxes to the sediments must most likely be derived from catchment inputs.

### 4.3 Climate Change and Environmental Implications

The results of this study suggest a strong relationship between climatic variation (1950–2016) and Hg flux ratios in the undisturbed lakes, especially since the mid-1990s. Since there has been no obvious increase in atmospheric Hg deposition, this implies that climate change has increased catchment erosion. Hg flux ratios declined during extreme dry events and increased during extreme wet events. Climate-change studies for the tropical and subtropical Americas have shown gradual or stepwise changes in temperature and extreme climate events since c. 2000.^66−68^ As indicated by SPEI data, analyses of long-term daily temperatures and precipitation time series for the 1961–2003 period from meteorological stations at the country level revealed a general warming trend in the region.^69^ Although no increase in annual rainfall amount has been reported, individual rainfall events, as observed in Gatun and Barbacoas lakes in 2010, appear to be intensifying in magnitude. A study by Angeles-Malaspina et al.^68^ demonstrated that annual maximum air temperatures, as derived from monthly averages of daily maximum air temperatures, increased in the intra-Americas at a rate of 0.006 °C per year between 1948 and 1998 (*p* = 0.02), but since 1998, this annual rate has increased to 0.03 °C (*p* = 0.01).

Changes in climate conditions such as drought, temperature, and flooding can affect catchment terrestrial ecosystems, weakening the stability of the catchment soils.^70−73^ Under drier conditions, increased temperatures can increase evaporation rates and reduce soil moisture. This results in reductions in plant biomass production and vegetation cover, and surface soils become fragile. Then, when rains come, and especially when heavy rainfall episodes occur, more soils could be eroded away, leading to increased soil erosion rates.^72^

Rapid increases in Hg fluxes to sediments of the undisturbed lakes, which exceed atmospheric Hg changes, suggest that climate change has passed a threshold that balanced catchment stability in some areas of the study region since c. 2000. The result has been an increase in soil erosion that brought more Hg stored in catchments into the lakes. Higher Hg ratios observed in sediments of our study lakes during extreme dry events are likely to arise from a two-phase Hg transport process. During initial drier periods, soil erosion rates increase because as soil moisture is reduced, plant growth and vegetation cover are negatively impacted.^11,73,74^ Subsequent episodic rains during wet seasons can wash greater amounts of terrestrial material into lakes.^11,75^ As a result, fluxes of Hg in sediments eventually increase as climate conditions become progressively drier. Increased temperatures after c. 2000 in this region correspond well with change points in the trend lines of Hg fluxes to the sediments in the undisturbed lakes (Figure 4b).

Extreme climate events can trigger immediate and time-lagged responses in ecosystems, and their effects on surface soils are nonlinear.^71^ Therefore, even small changes in the frequency or severity of climate extremes could considerably affect soil stability and erosion. Mean temperatures in Central and South America will continue to increase.^76^ Mean precipitation is projected to increase or decrease in different subregions of South America, while tropical cyclones with higher precipitation, severe storms, and dust storms are expected to become more frequent in the Caribbean and Northern and Southern Central America, and extremely high temperatures are expected to increase.^76^ With anticipated future climate-change scenarios for this region,^15^ continued increases in Hg fluxes to sediments from catchments are likely to occur.

Based on high-resolution rainfall records, a soil-erosion assessment suggests that South America and the Caribbean countries are among the areas that have the highest erosivity values in the world.^77^ This implies that, compared with remote lakes in some other regions with relatively low erosivity values such as Alaska,^4,77^ lakes in the tropical and subtropical American region may be more sensitive to soil-erosion effects arising from climate change.

If climatic changes continue at their current rate, the stabilities of lake and catchment ecosystems are likely to be altered, and their structures and functions will change. Progressively, the thresholds of catchment soil stability are likely to be exceeded worldwide, which would result in increased inputs to lakes of contaminants including Hg. A general increase in Hg fluxes to lake sediments in many regions may not be far away. Lake-sediment records have been used intensively in the past to reveal atmospheric pollution histories, but changes to catchment soils could introduce challenges for that approach in the future.

## Supporting Information Available

Sediment radiometric chronologies and sedimentation rates of the study cores (Figures S1–S23); SPEI analyses showing climate tendencies in individual sites and the relations with Hg flux ratios to the sediments (Figures S24 and S25); chemical element distribution in the Yojoa core taken from Yojoa Lake, Honduras (Figure S26); mercury concentrations in the sediments formed since the 1950s in the study sediment cores (Figure S27); possible different environmental settings of the study sites (Figure S28); total SO_2_ emissions from volcanic eruption in the study region (Table S1 and Figure S29); mercury concentrations versus time in the sediment cores from the study sites (Figure S30); mercury concentrations in the sediment cores (Table S2); ^210^Pb chronologies and sedimentation rates in the sediment cores (Tables S3–S13); mercury fluxes in the sediment cores (Table S14); mercury flux ratios (against 1850 values, except the cores from lakes Yojoa, Gatun, and Barbacoas) versus time in the sediment cores from the study sites (Table S15); how increased anthropogenic Hg emissions could affect Hg fluxes into the lake sediments (Table S16); references (PDF)
