# Supplementary material for: Mercury Pollution History in Tropical and Subtropical American Lakes: Multiple Impacts and the Possible Relationship with Climate Change
Source: Environ Sci Technol. 2023 Feb 21;57(9):3680–90. doi: 10.1021/acs.est.2c09870 (PMC9996825; doi:10.1021/acs.est.2c09870)
Supplement: Supplementary file 1 — es2c09870_si_001.pdf [file es2c09870_si_001.pdf]

Supporting Information

Mercury pollution history in tropical and subtropical American lakes: multiple impacts and the possible relationship with climate change

Handong Yang [a, *], Laura Macario-González [b,c], Sergio Cohuo [b,d], Thomas J. Whitmore [e], Jorge Salgado [a,f,g,h], Liseth Peréz [b], Antje Schwalb [b], Neil L Rose [a], Jonathan Holmes [a], Melanie A. Riedinger-Whitmore [e], Philipp Hoelzmann [i], Aaron O'Dea [h]

[a] Environmental Change Research Centre, University College London, Gower Street, London WC1E 6BT, UK

[b] Institut für Geosysteme und Bioindikation, Technische Universität Braunschweig, D-38106 Braunschweig, Langer Kamp 19c, Germany

[c] Tecnológico Nacional de México – I. T. de la Zona Maya, Carretera Chetumal-Escárcega Km 21.5, Ejido Juan Sarabia, 77965 Quintana Roo, Mexico

[d] Tecnológico Nacional de México – I. T. Chetumal., Av. Insurgentes 330, Chetumal, 77013 Quintana Roo, Mexico

[e] University of South Florida, 140 7th Avenue South, St. Petersburg, Florida 33701, USA

[f] Programa de Ingeniería Civil, Grupo de Infraestructura y Desarrollo Sostenible, Universidad Católica de Colombia, Bogotá, 111311, Colombia

[g] School of Geography, University of Nottingham, Nottingham, NG7 2RD, UK

[h] Smithsonian Tropical Research Institute, PO Box 0843-03092, Balboa, Panama

[i] Institut für Geographische Wissenschaften, Freie Universität Berlin, D-12249 Berlin, Malteser Strasse 74-100, Germany

This Supporting Information has 35 pages including 30 figures and 16 tables.

Contents:

1. **Sediment radiometric chronologies and sedimentation rates of the study cores (Figures S1 – S23).**

2. **SPEI analyses show climate tendencies in individual sites and the relations with Hg Flux rations to the sediments (Figure S24 – S25).**

3. **Chemical element distribution in the in the Yojoa core taken from Yojoa Lake, Honduras (Figure S26).**

4. **Mercury concentrations in the sediments formed since the 1950s in the study sediment cores (Figure S27).**

5. **Possible different environmental settings of the study sites (Figure S28).**

6. **Total SO_2 emissions from volcanic eruption in the study region (Table S1, Figure S29).**

7. **Mercury concentrations versus time in the sediment cores from the study sites (Figure S30).**

8. **Mercury concentrations in the sediment cores (Table S2).**

9. **^{210}Pb chronologies and sedimentation rates in the sediment cores (Table S3 – S13).**

10. **Mercury fluxes in the sediment cores (Table S14).**

11. **Mercury flux ratios (against 1850 values, except the cores from lakes Yojoa, Gatun and Barbacoas) versus time in the sediment cores from the study sites (Table S15).**

12. **How increased anthropogenic Hg emissions could affect Hg fluxes into the lake sediments (Table S16).**

13. **References for SI**

1. Sediment radiometric chronologies of the study cores

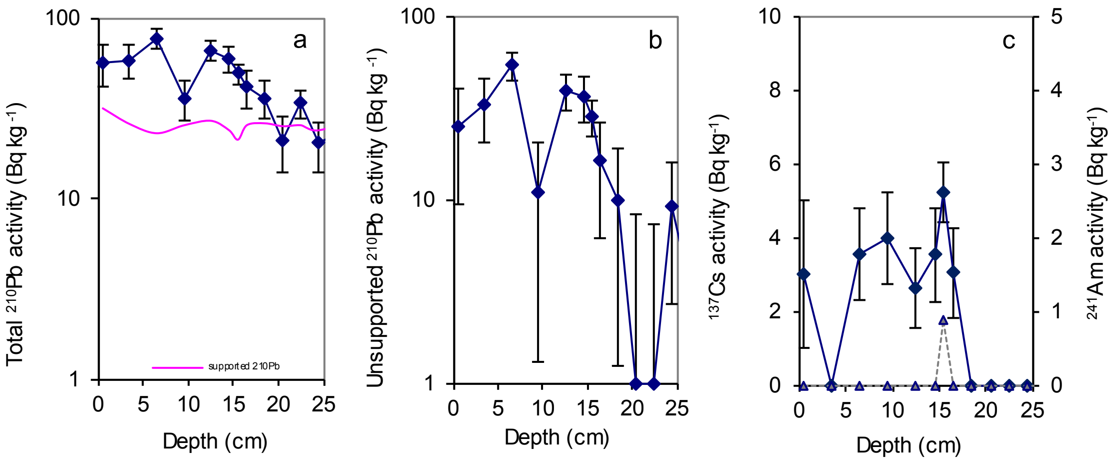

Figure S1. Fallout radionuclide concentrations in core BFWP taken from a Barbuda freshwater pond, showing (a) total ^{210}Pb, (b) unsupported ^{210}Pb, (c) ^{137}Cs (lozenge symbol) and ^{241}Am (triangle symbol) concentrations versus depth.

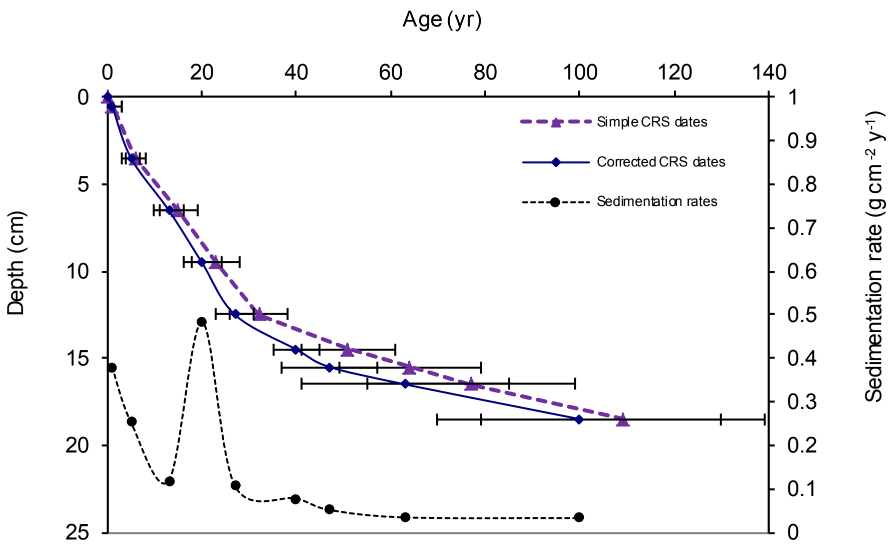

Figure S2. Radiometric chronology of core BFWP taken from a Barbuda freshwater pond, showing the CRS model ^{210}Pb dates and sedimentation rates (Appleby, 2000).

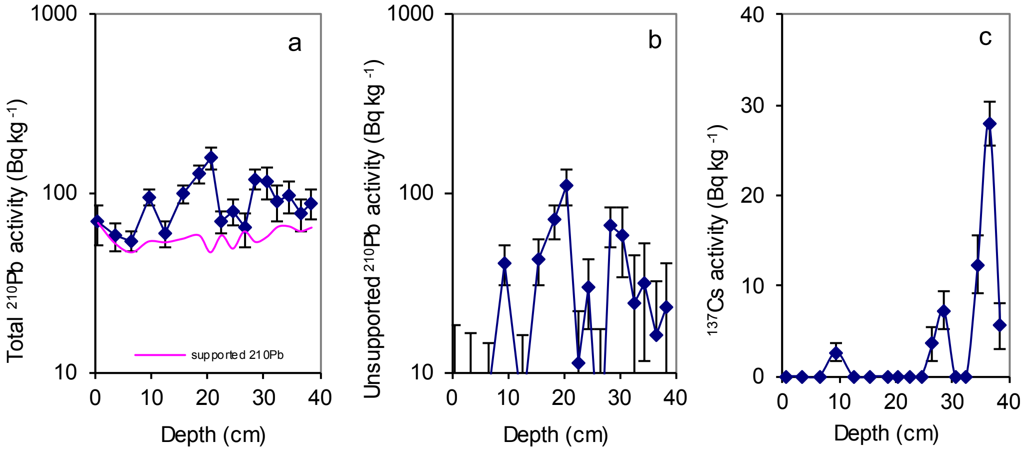

Figure S3. Fallout radionuclide concentrations in core LBARB1 taken from Lake Barbacoas, showing (a) total ^{210}Pb, (b) unsupported ^{210}Pb, and (c) ^{137}Cs concentrations versus depth.

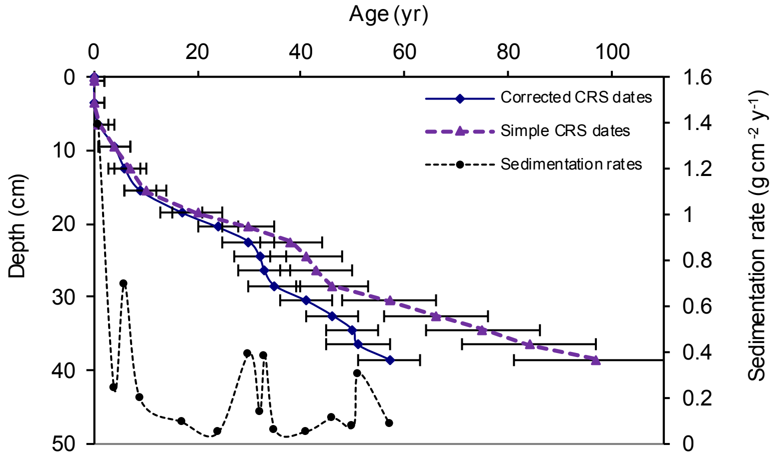

Figure S4. Radiometric chronology of core LBARB1 taken from Lake Barbacoas, showing the CRS model ^{210}Pb dates and sedimentation rates.

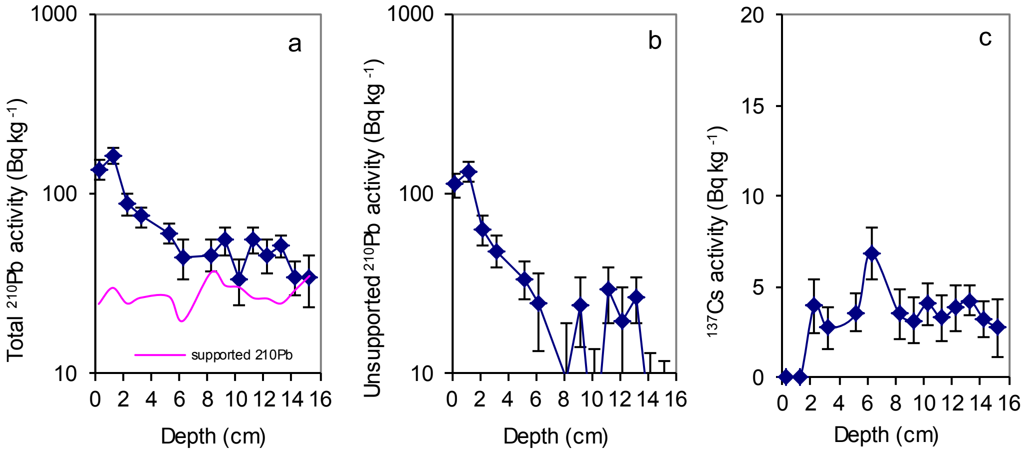

Figure S5. Fallout radionuclide concentrations in core APAS taken from Apastepeque, showing (a) total ^{210}Pb, (b) unsupported ^{210}Pb and (c) ^{137}Cs concentrations versus depth.

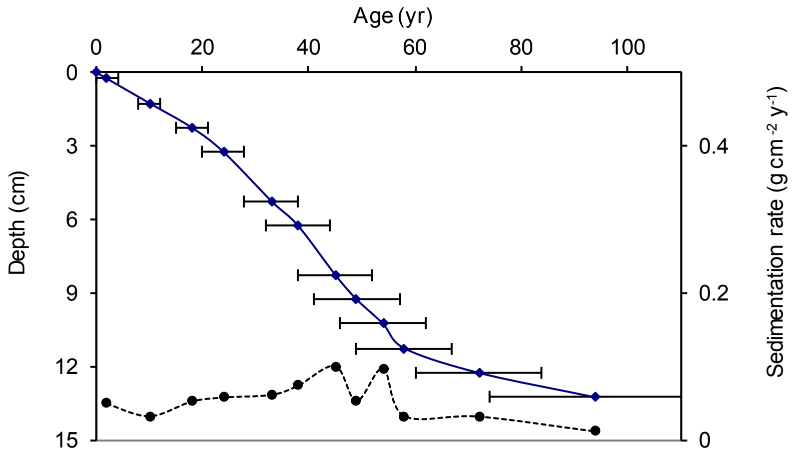

Figure S6. Radiometric chronology of core APAS taken from Apastepeque, showing the CRS model ^{210}Pb dates and accumulation rates. The solid line shows age while the dashed line indicates accumulation rate.

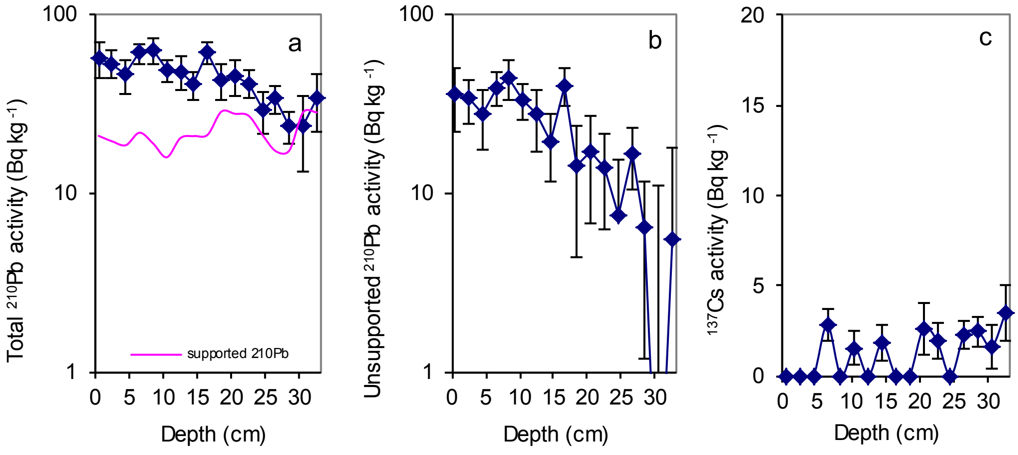

Figure S7. Fallout radionuclide concentrations in core OLOM taken from Laguna Olomega, Salvador, showing (a) total ^{210}Pb, (b) unsupported ^{210}Pb, and (c) ^{137}Cs concentrations versus depth.

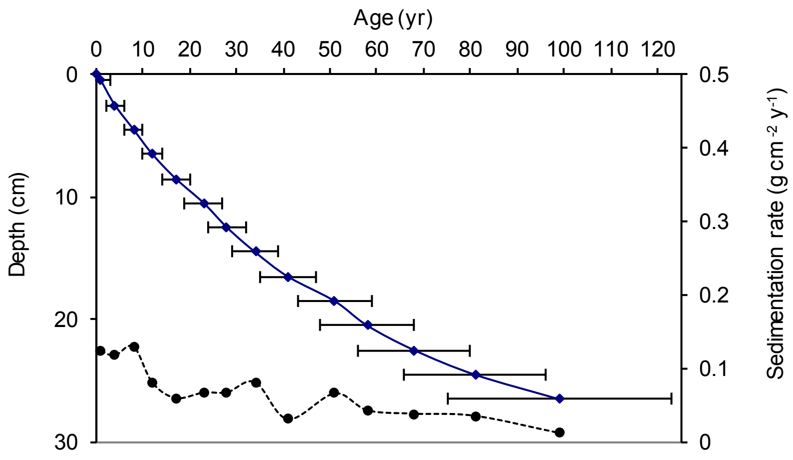

Figure S8. Radiometric chronology of core OLOM taken from Laguna Olomega, Salvador, showing the CRS model ^{210}Pb dates and sedimentation rates. The solid line shows age while the dashed line indicates sedimentation rate.

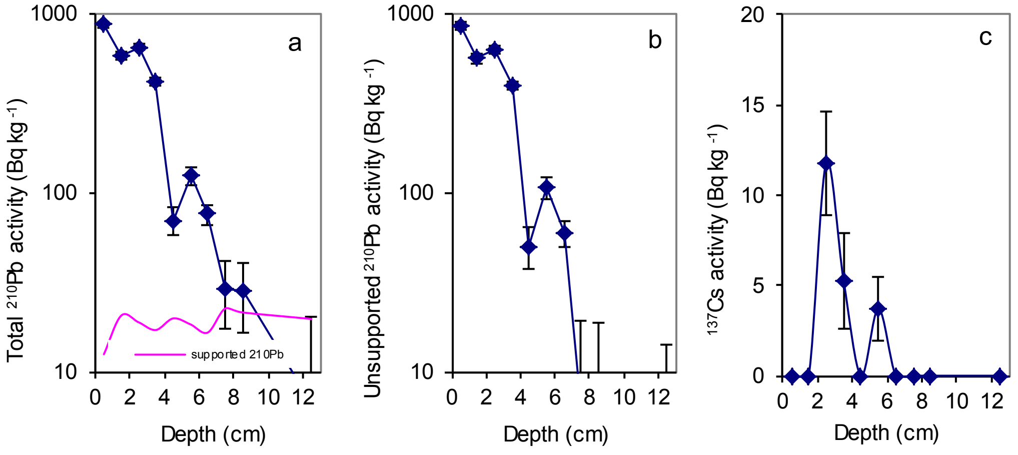

Figure S9. Fallout radionuclide concentrations in core VERDE taken from Laguna Verde, Salvador, showing (a) total ^{210}Pb, (b) unsupported ^{210}Pb, and (c) ^{137}Cs concentrations versus depth.

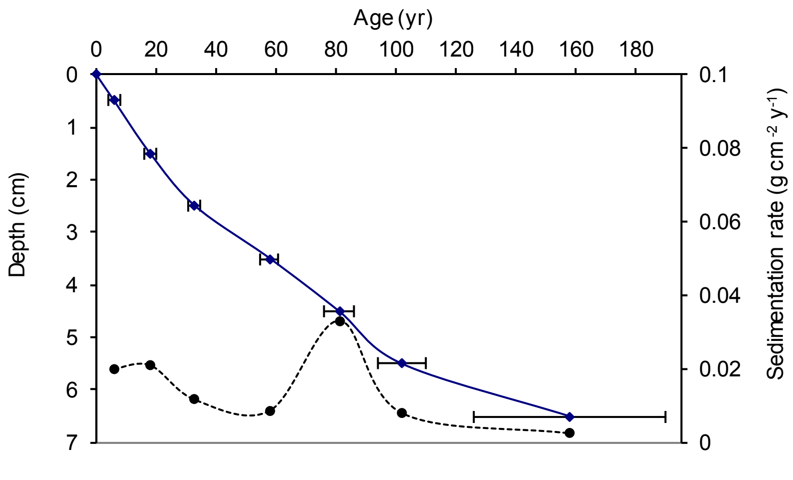

Figure S10. Radiometric chronology of core VERDE taken from Laguna Verde, Salvador, showing the CRS model ^{210}Pb dates and sedimentation rates. The solid line shows age while the dashed line indicates sedimentation rate.

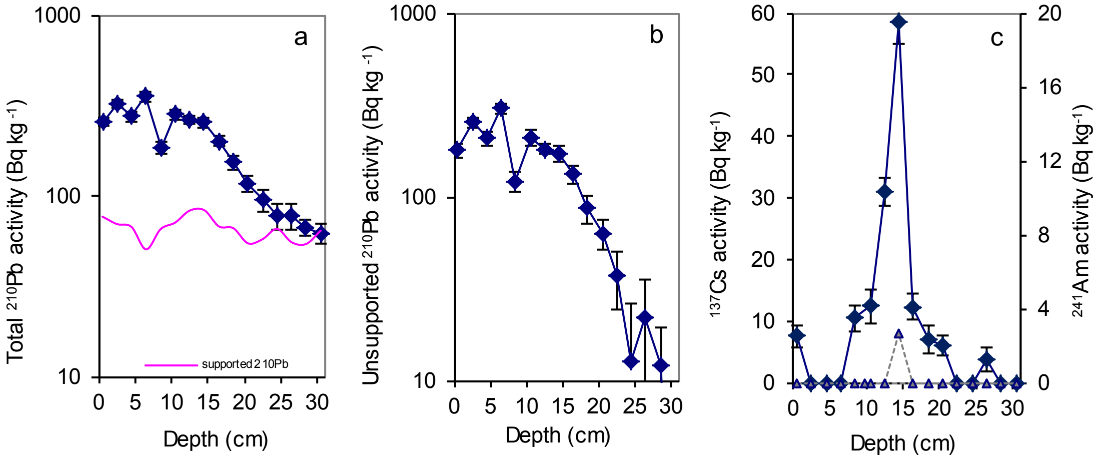

Figure S11. Fallout radionuclide concentrations in core YOJOA taken from Lake Yojoa, Honduras, showing (a) total ^{210}Pb, (b) unsupported ^{210}Pb, and (c) ^{137}Cs (lozenge) and ^{241}Am (triangle) concentrations versus depth.

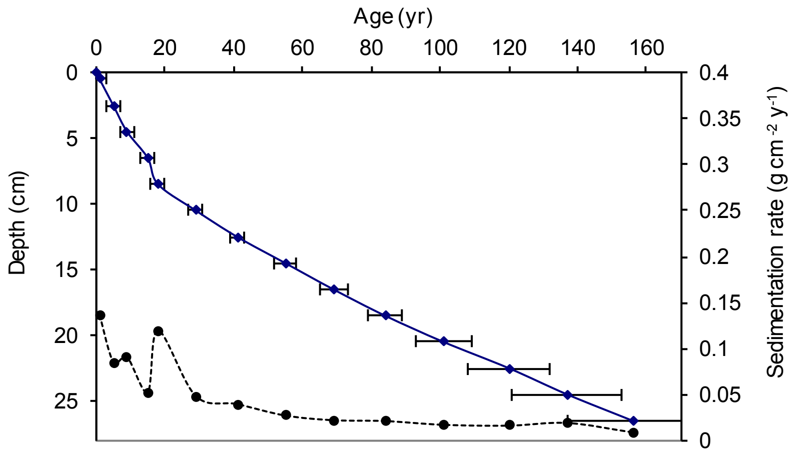

Figure S12. Radiometric chronology of core YOJOA taken from Lake Yojoa, Honduras, showing the CRS model ^{210}Pb dates and sedimentation rates. The solid line shows age while the dashed line indicates sedimentation rate.

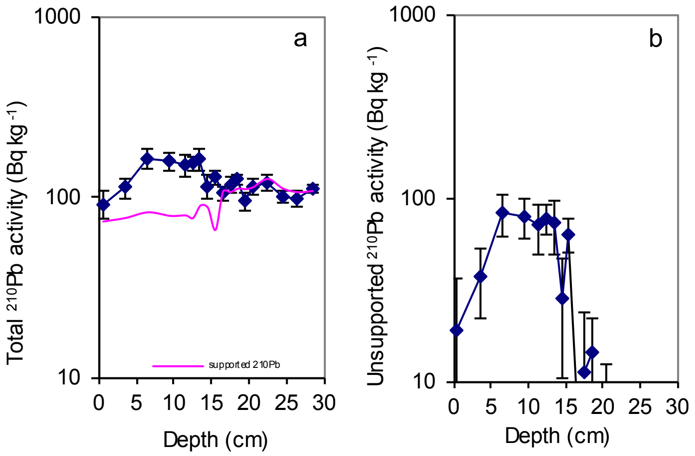

Figure 13. Fallout radionuclide concentrations in core WAPG1 taken from Wallywash great Pond, Jamaica, showing (a) total ^{210}Pb and (b) unsupported ^{210}Pb concentrations versus depth.

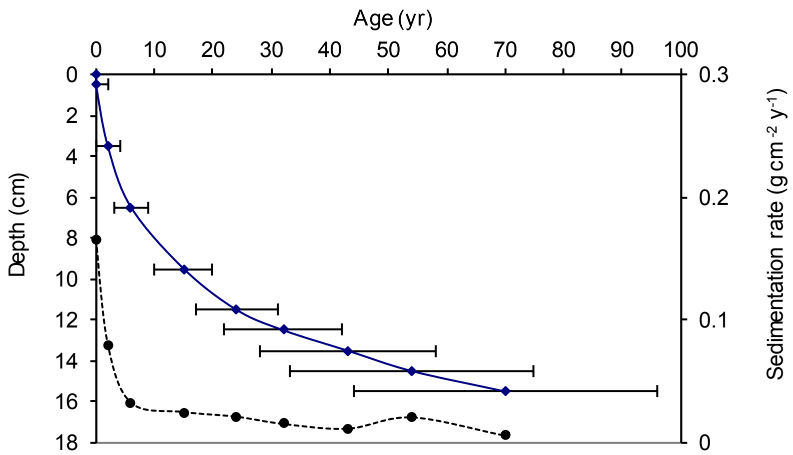

Figure 14. Radiometric chronology of core WAPG1 taken from Wallywash Great Pond, Jamaica, showing the CRS model ^{210}Pb dates and sedimentation rates. The solid line shows age while the dashed line indicates sedimentation rate.

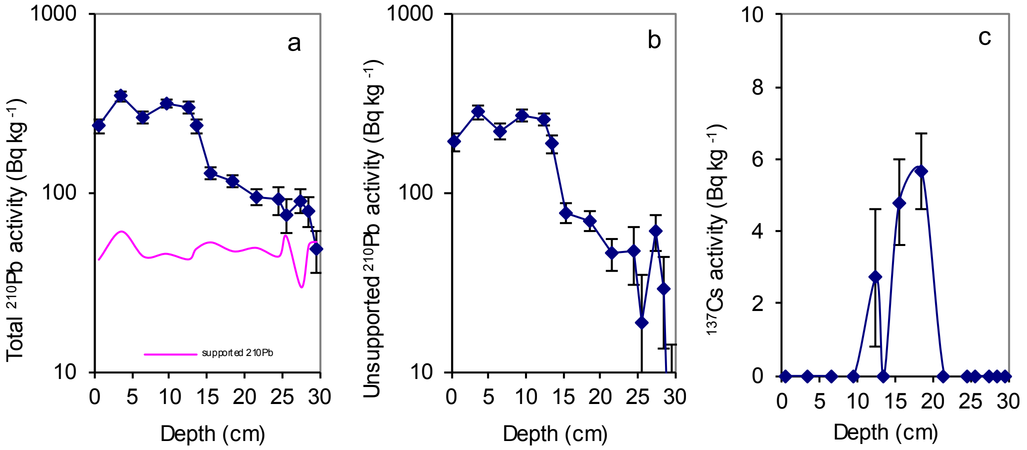

Figure S15. Fallout radionuclide concentrations in core YCH taken from Yaal Chac, Maxico, showing (a) total ^{210}Pb, (b) unsupported ^{210}Pb and (c) ^{137}Cs concentrations versus depth.

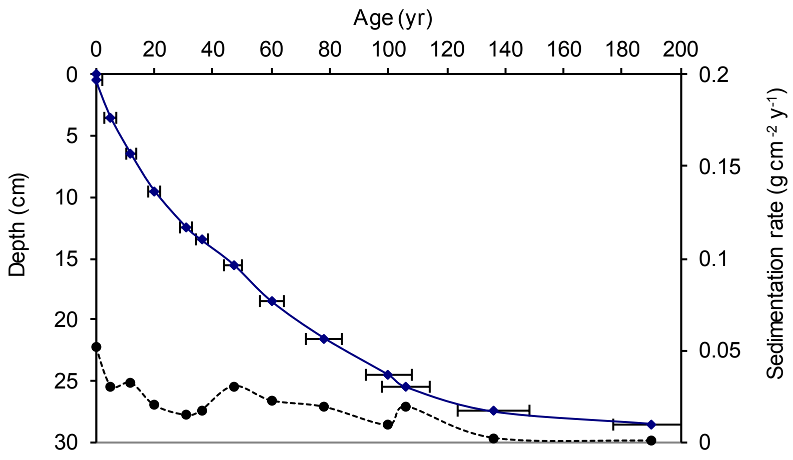

Figure S16. Radiometric chronology of core YCH taken from Yaal Chac, Maxico, showing the CRS model ^{210}Pb dates and sedimentation rates. The solid line shows age while the dashed line indicates sedimentation rate.

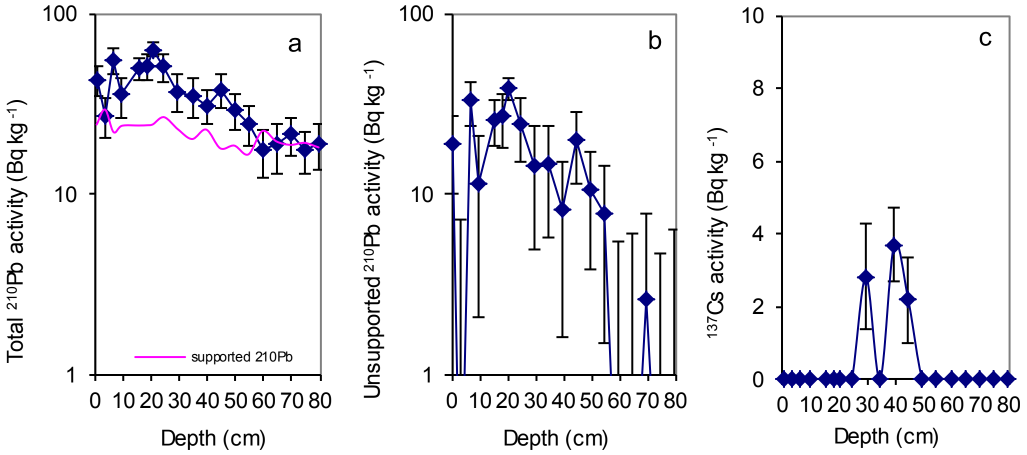

Figure S17. Fallout radionuclide concentrations in core LGAT1 taken from Gatun Lake, showing (a) total ^{210}Pb, (b) unsupported ^{210}Pb and (c) ^{137}Cs concentrations versus depth.

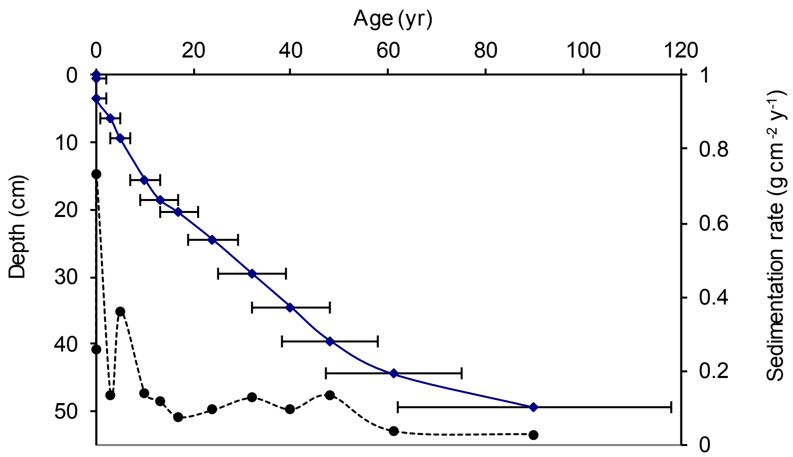

Figure S18. Radiometric chronology of core LGAT1 taken from Gatun, showing the CRS model ^{210}Pb dates and sedimentation rates. The solid line shows age while the dashed line indicates sedimentation rate.

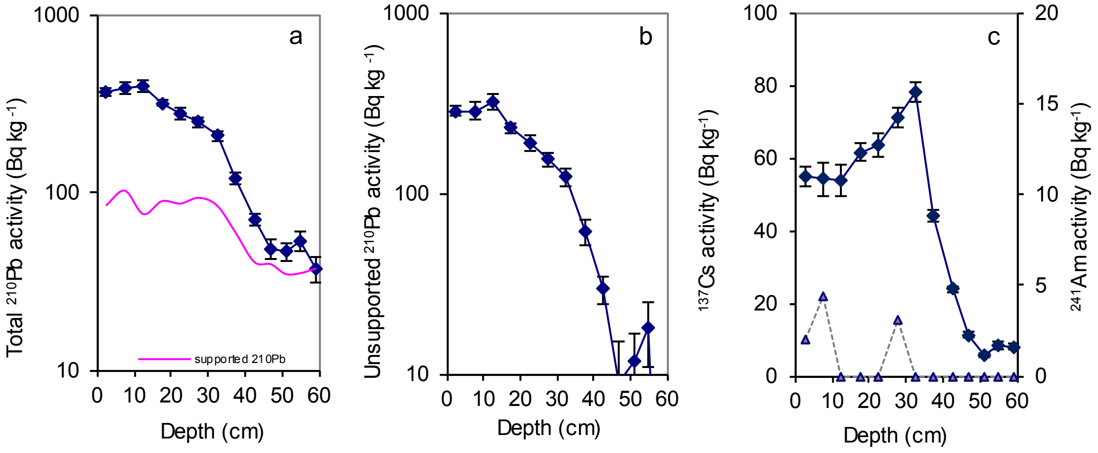

Figure S19. Fallout radionuclide concentrations in core CY40 taken from Cypress, showing (a) total ^{210}Pb, (b) unsupported ^{210}Pb, and (c) ^{137}Cs (lozenge) and ^{241}Am (triangle) concentrations versus depth.

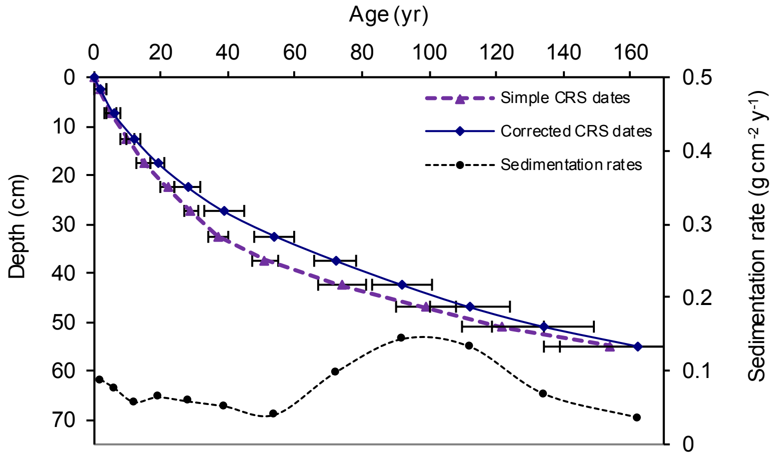

Figure S20. Radiometric chronology of core CY40 taken from Cypress, showing the CRS model ^{210}Pb dates and sedimentation rates.

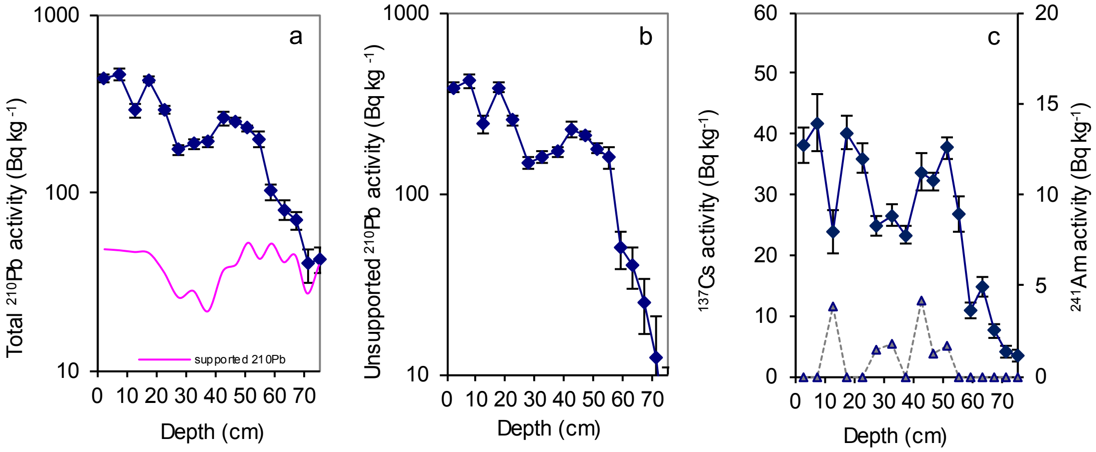

Figure S21. Fallout radionuclide concentrations in core KIS43 taken from Kissimmee, showing (a) total ^{210}Pb, (b) unsupported ^{210}Pb, and (c) ^{137}Cs (lozenge) and ^{241}Am (triangle) concentrations versus depth.

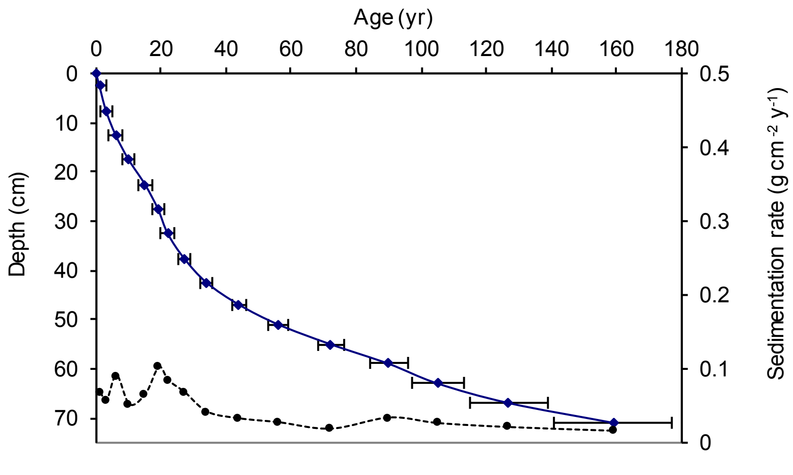

Figure S22. Radiometric chronology of core KIS43 taken from Kissimmee, showing the CRS model ^{210}Pb dates and sedimentation rates. The solid line shows age while the dashed line indicates sedimentation rate.

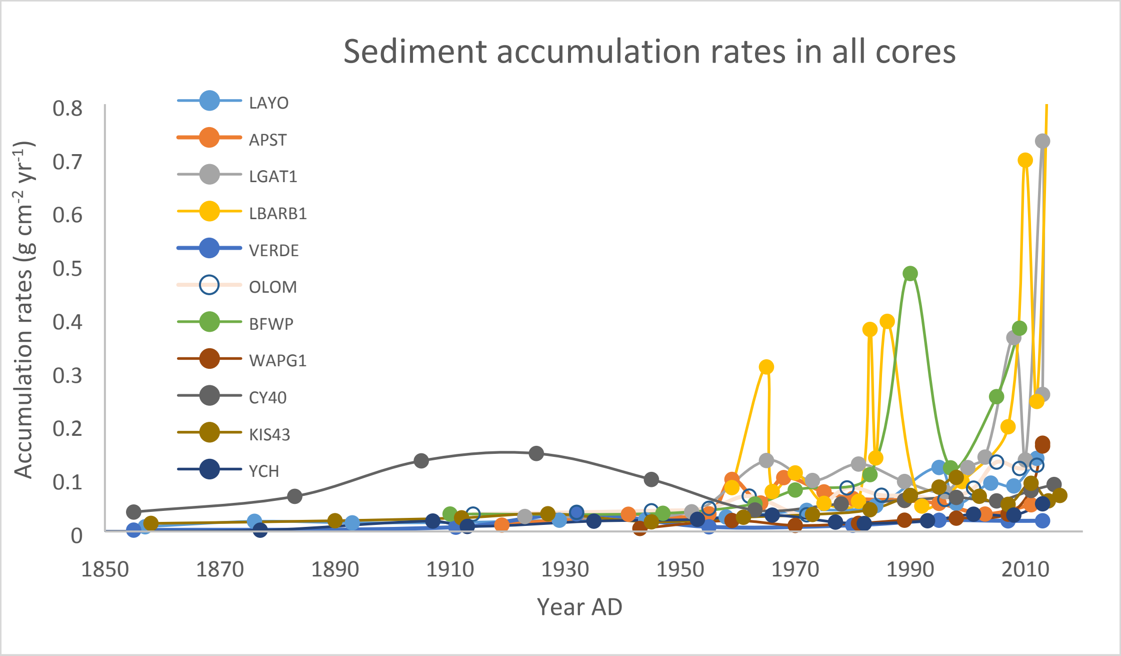

Figure S23. Sediment accumulation rates in all the study cores.

2. **SPEI analyses show climate tendencies in individual sites and the relations with Hg flux rations to the sediments.**

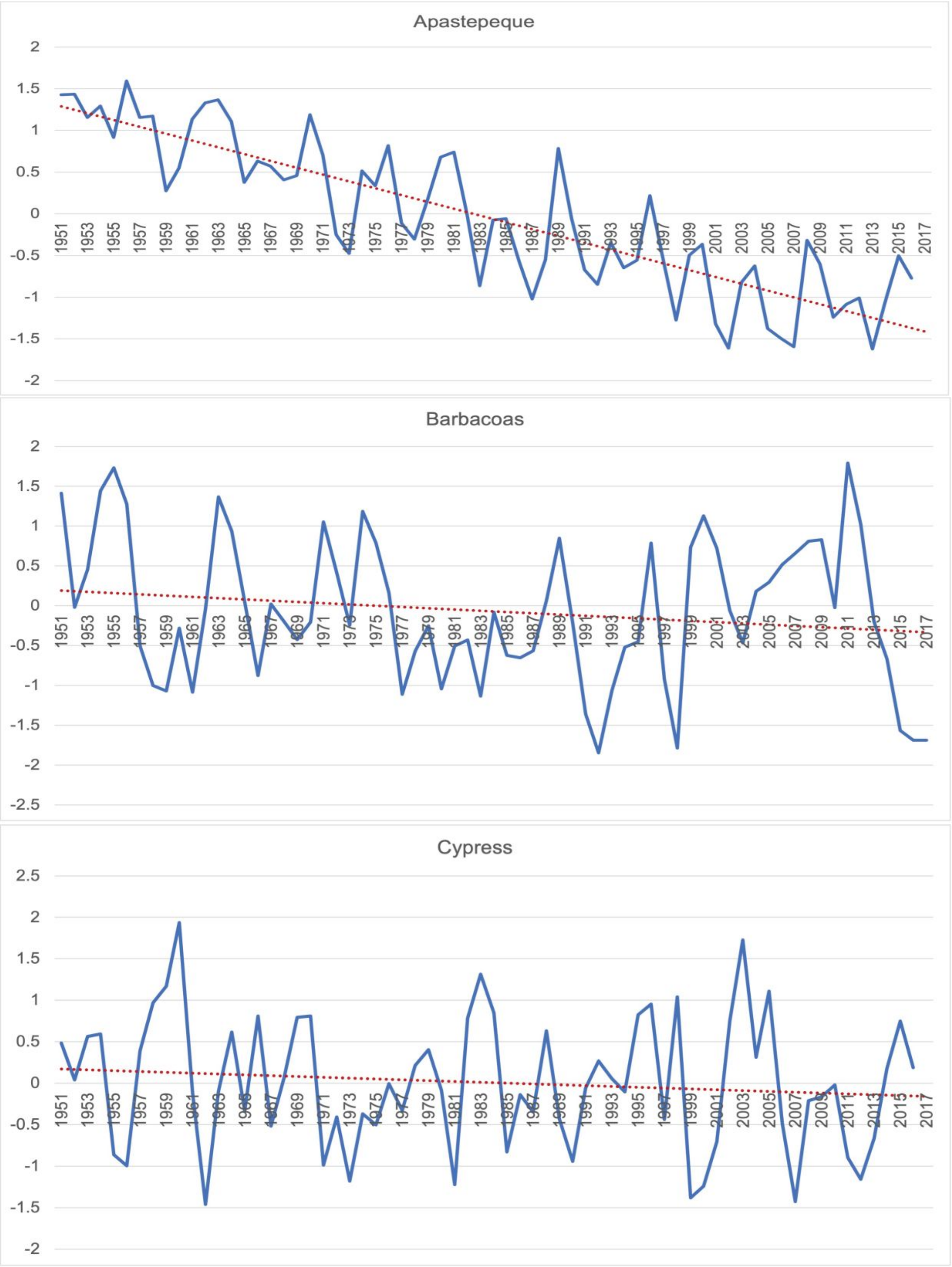

Figure S24. The SPEI analyses show that tendencies in most sites tend to drier climates with time during 1951 – 2017. Positive SPEI value suggests wet while negative indicates dry with respect to normal conditions.

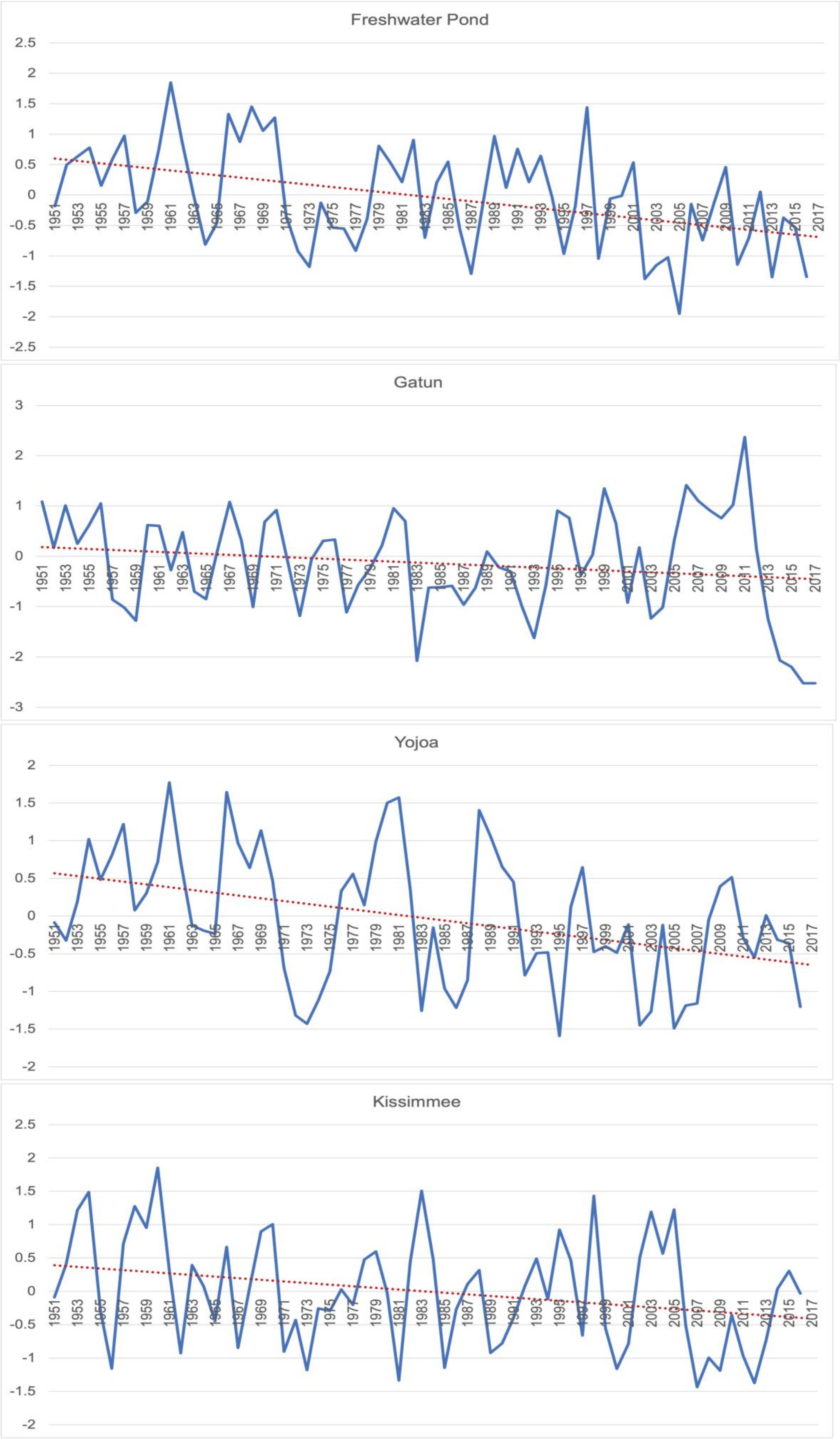

Figure S24. C*ontinued*.

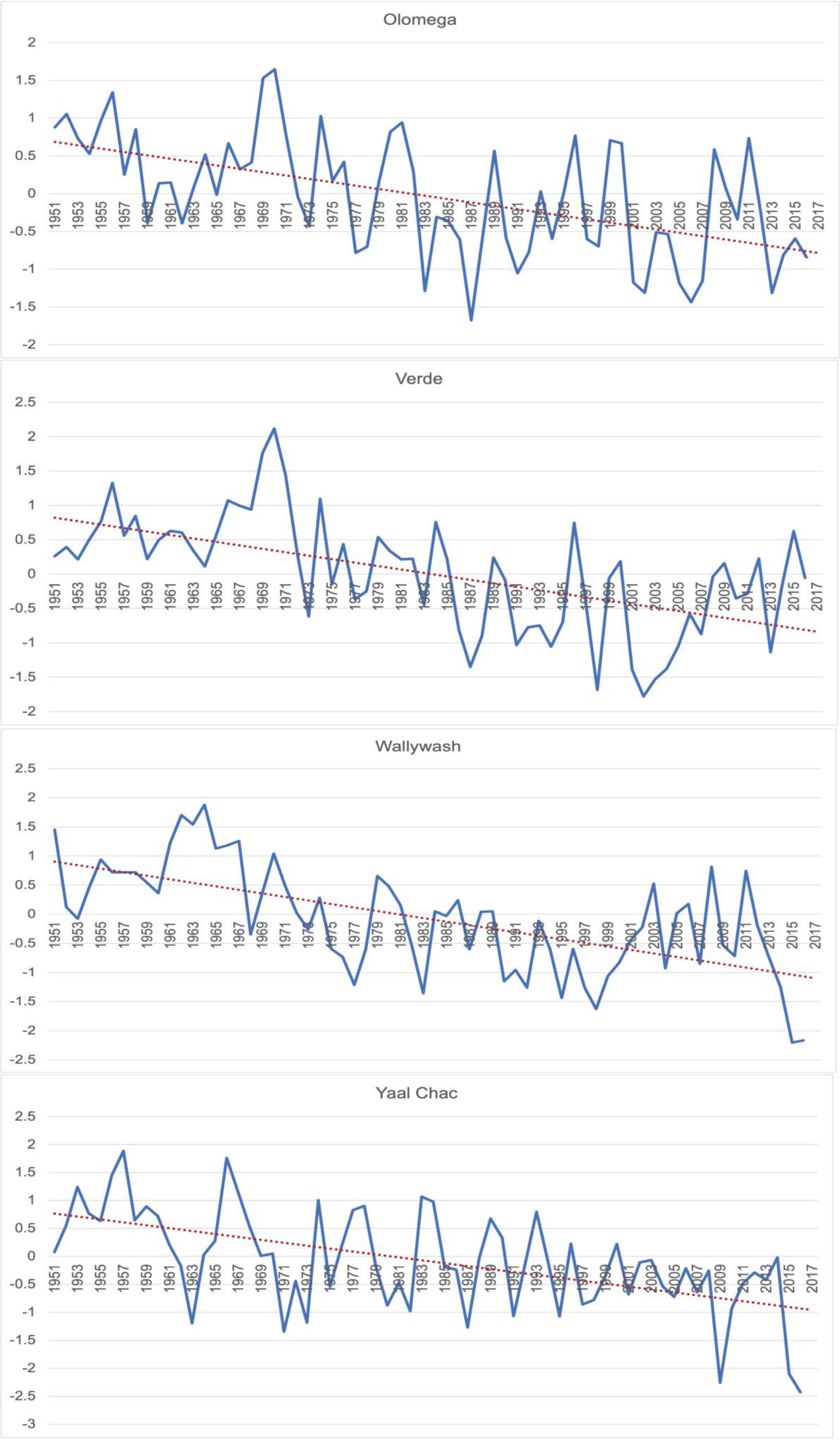

Figure S24. *Continued.*

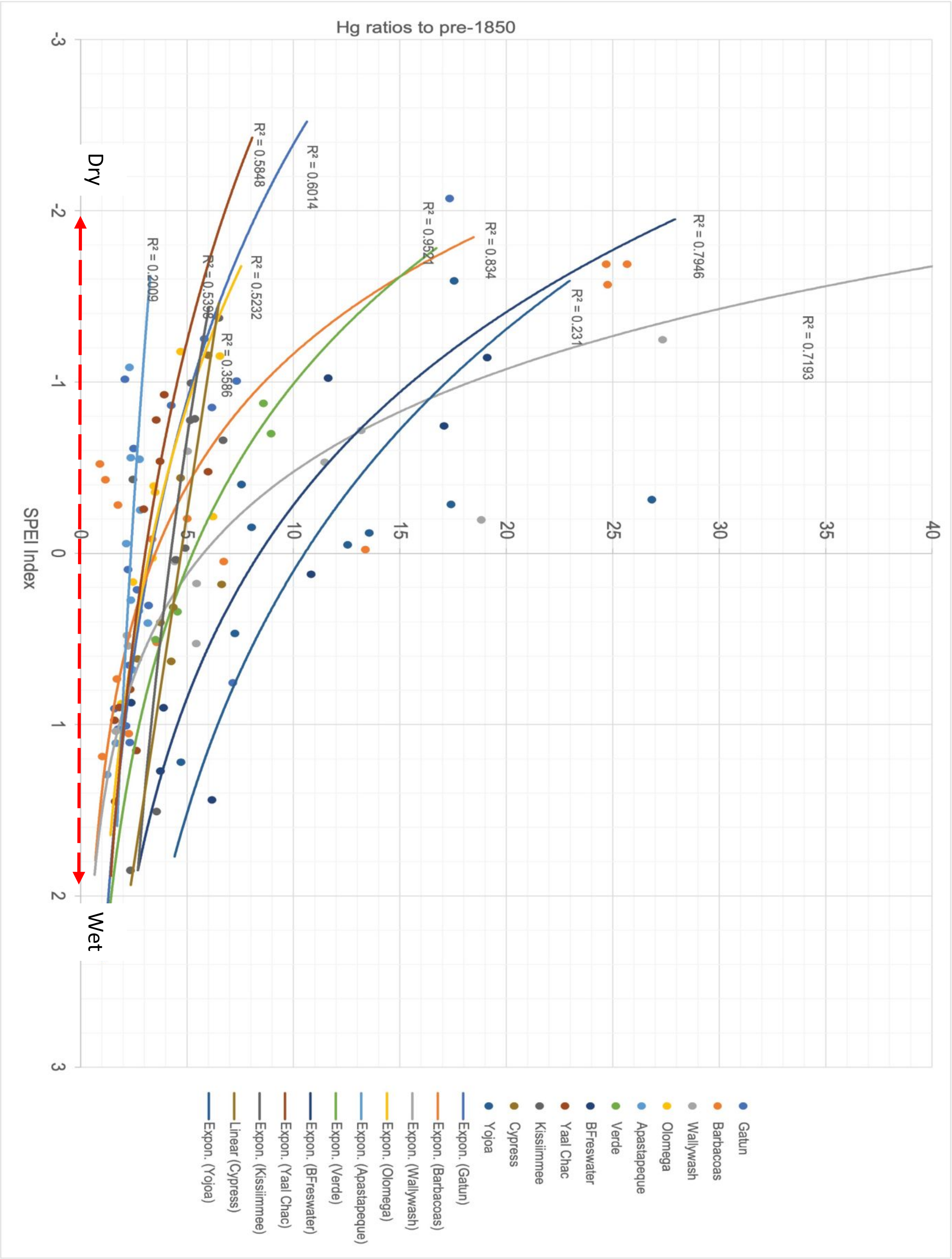

Figure S25. Corrections between Hg flux ratios and SPEI indexes. SPEI index values < -2 are considered to be extremely dry, -2 to -0.5 dry; SPEI indexes in 0.5 to 2 are considered as wet, and > 2 extremely wet (also see the manuscript text).

3. Chemical element distribution in the in the Yojoa core taken from Yojoa Lake, Honduras

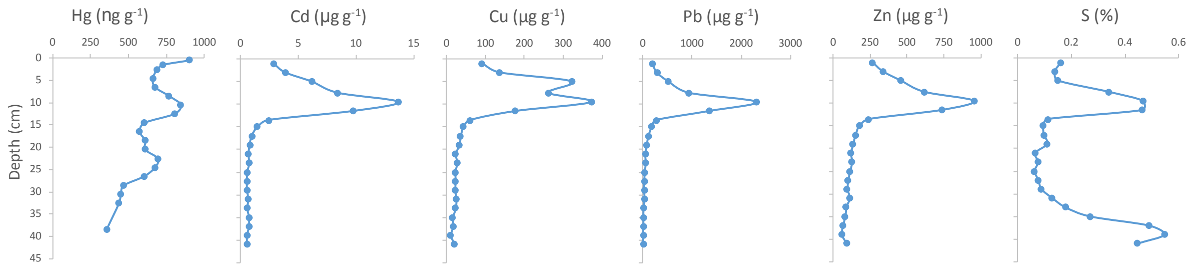

Figure S26. Distribution of chemical elements in the Yojoa core taken from Yojoa Lake, Honduras.

4. Mercury concentrations in the sediments formed since the 1950s in the study sediment cores

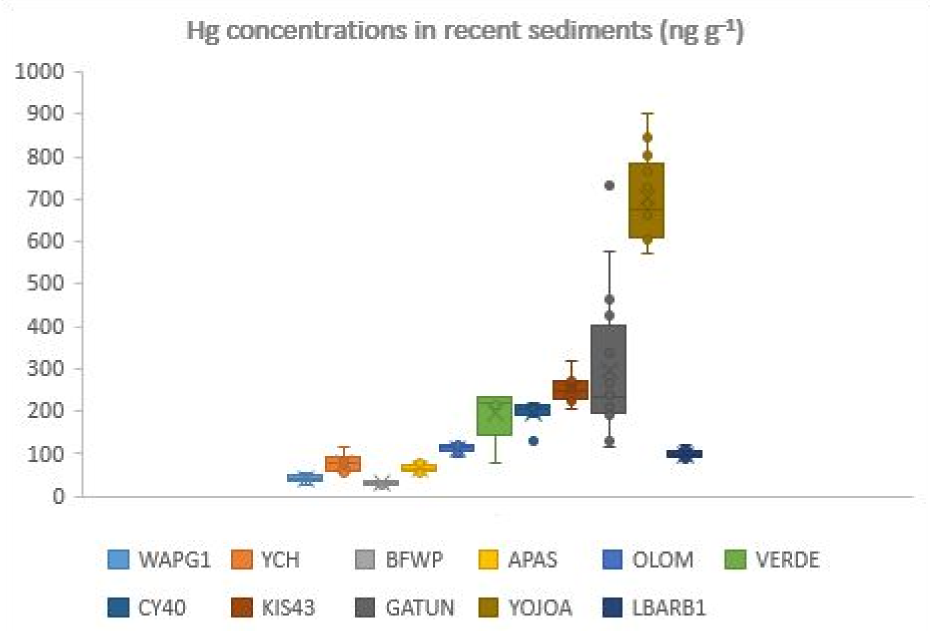

Figure S27. Mercury cocentrations in the sediments formed since the 1950s in the study sediment cores.

The cores WAPG1, YCH, BFWP, OLOM and VERDE are from pristine sites, they generally have lower Hg concentrations (mostly in 40 – 100 ng g-1 in modern sediments) than those in the lakes that have been affected by local sources. Hg concentrations are over 200 ng g-1 in the sediments formed after the 1950s in the VERDE core, which might be caused by slow sedimentation rates as 5 cm surface sediments cover about last hundred years and deposited anthropogenic Hg possibly concentrated in the surface sediments in this closed crater lake. The cores from the sites that have been affected by local human activities, normally have relatively high Hg concentrations. However, the Hg concentrations in LBARB1 are relatively low simlar with those from pristine sites, it is because Lake Barbacoas has been affected by deforestion and the sedimentation rates have massively increased (Figure S4) that diluted Hg concentrations in the sediments. Core APAS from Apastepeque show low Hg concnetrations although managed cultivation has occurred in the catchment in recent years.

5. Possible different environmental settings of the study sites

When atmospheric Hg deposited into a lake’s terrestrial catchment surface, part of the deposited Hg is absorbed to the catchment surface soils or plants, while the other marks its way to the lake. In a region, if environmental settings of lake sites are similar, for example, the catchment slopes, vegetation coverages, soils types and coverages, etc., are similar, the fractions of atmospherically deposited Hg that deposited to the catchment and then washed into the lake can be similar (Swain et al., 1992; Yang, 2015; Drevnick et al., 2016). The relationship of Hg accumulation rates with the ratios of catchment area to lake area in all the study sites show poor relations in all the specific times (Figure S24: 1850-all, 1990-all and 2010-all), suggesting catchment inputs to the lakes vary among the sites in these specific times. This is because these sites have different Hg sources. While some sites have anthropogenic Hg source only through deposition, others have affected by local human activities such as mining, dam construction, local human settlement and industry development. Part of the sites, which receive anthropogenic Hg possibly only from deposition, also have poor relations between Hg accumulation rates and the ratios of catchment area to lake area (1850-part, 1990-part, 2010-part), this suggests that the catchment settings among these sites are considerably different, possibly due to different catchment slopes, vegetation coverages, soils types and coverages, etc., which results in different fractions of atmospherically deposited Hg to the catchment to be transported into the lakes. However, the patterns of 1850-part and 1990-part are similar, may imply that the fractions of atmospherically deposited Hg to the catchment to be in-washed into the individual lakes maybe different but have not been changed much. Which means the environmental setting in these individual sites have not changed considerably in the 1850s and the 1990s. The pattern of 2010-part is different from those of 1850-part and 1990-part, suggesting the environmental settings may be different from those in the 1850s and 1990s, which means environmental settings in those lake sites have changed in recent one or two decades.

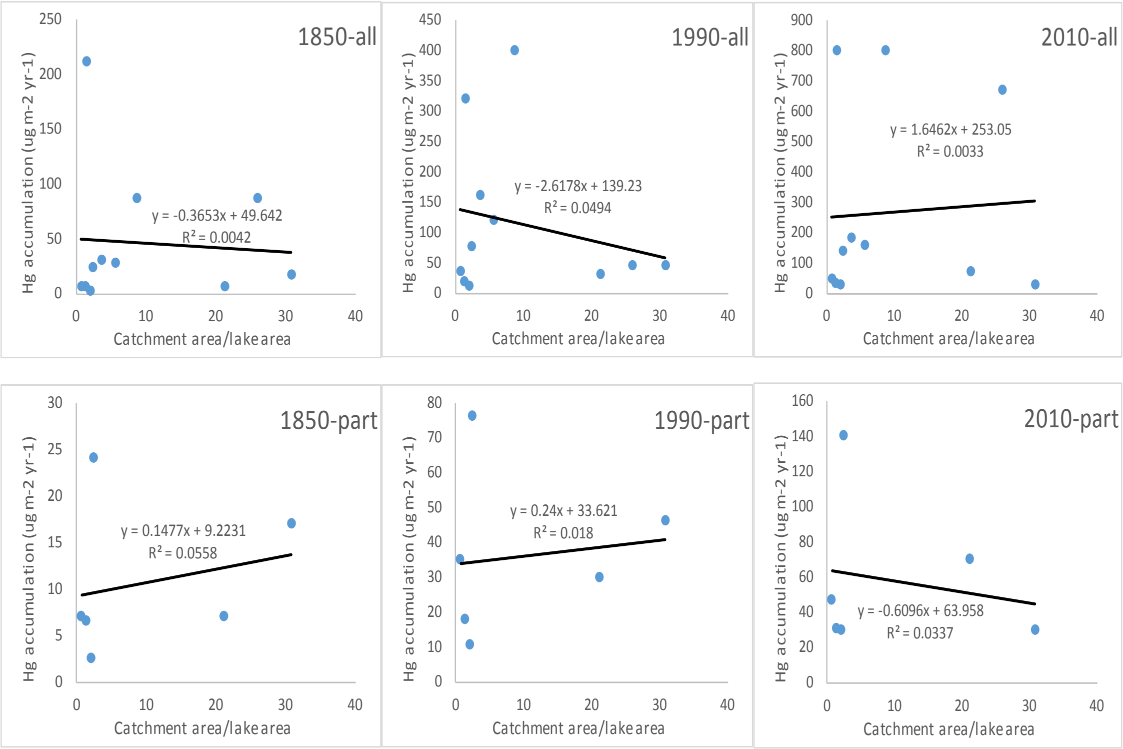

Figure S28. Relationships of Hg accumulation in the lake sediments with the ratios of catchment area to lake areas.

6. Total SO_2 emissions from volcanic eruption in the study region

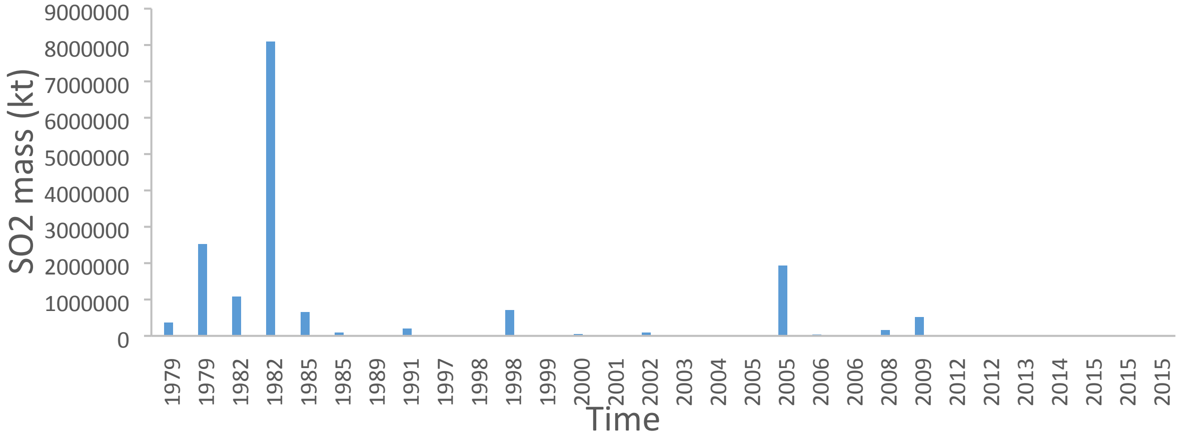

Figure S29. Total SO2 emissions from volcanic eruption (≥ 10 kt, at least 15 km altitude) detected using satellite-based instruments in the study region (data listed in Table 1).

Table S1. Total SO_2 emissions from volcanic eruption (≥ 10 kt, at least 15 km altitude) detected using satellite-based instruments in the study region as shown in Figure 1.

Volcanos	Eruption year	SO2 mass (kt)
Telica	2015	1000
momotombo	2015	1500
Cotopaxi	2015	20000
Turrialba	2014	3000
San Miguel	2013	5000
San Cristobal (Nicaragua)	2012	5000
San Cristobal (Nicaragua)	2012	1000
Fernandina	2009	521000
Azul, Cerro	2008	157000
Tungurahua	2006	10000
Tungurahua	2006	30000
Negra, Sierra	2005	1928000
Santa Ana	2005	12000
Galeras	2004	3000
Tungurahua	2003	12000
Reventador	2002	84000
Popocatepetl	2001	4000
Popocatepetl	2000	49000
Tungurahua	1999	6000
Azul, Cerro	1998	714000
Popocatepetl	1998	10000
Popocatepetl	1997	7000
Marchena	1991	200000
Ruiz, Nevado del	1989	12000
Ruiz, Nevado del	1985	90000
Ruiz, Nevado del	1985	660000
Chichon, El	1982	8090000
Wolf	1982	1080000
Negra, Sierra	1979	2524000
Azul, Cerro	1979	358000

7. Mercury concentrations versus time in the sediment cores from the study sites.

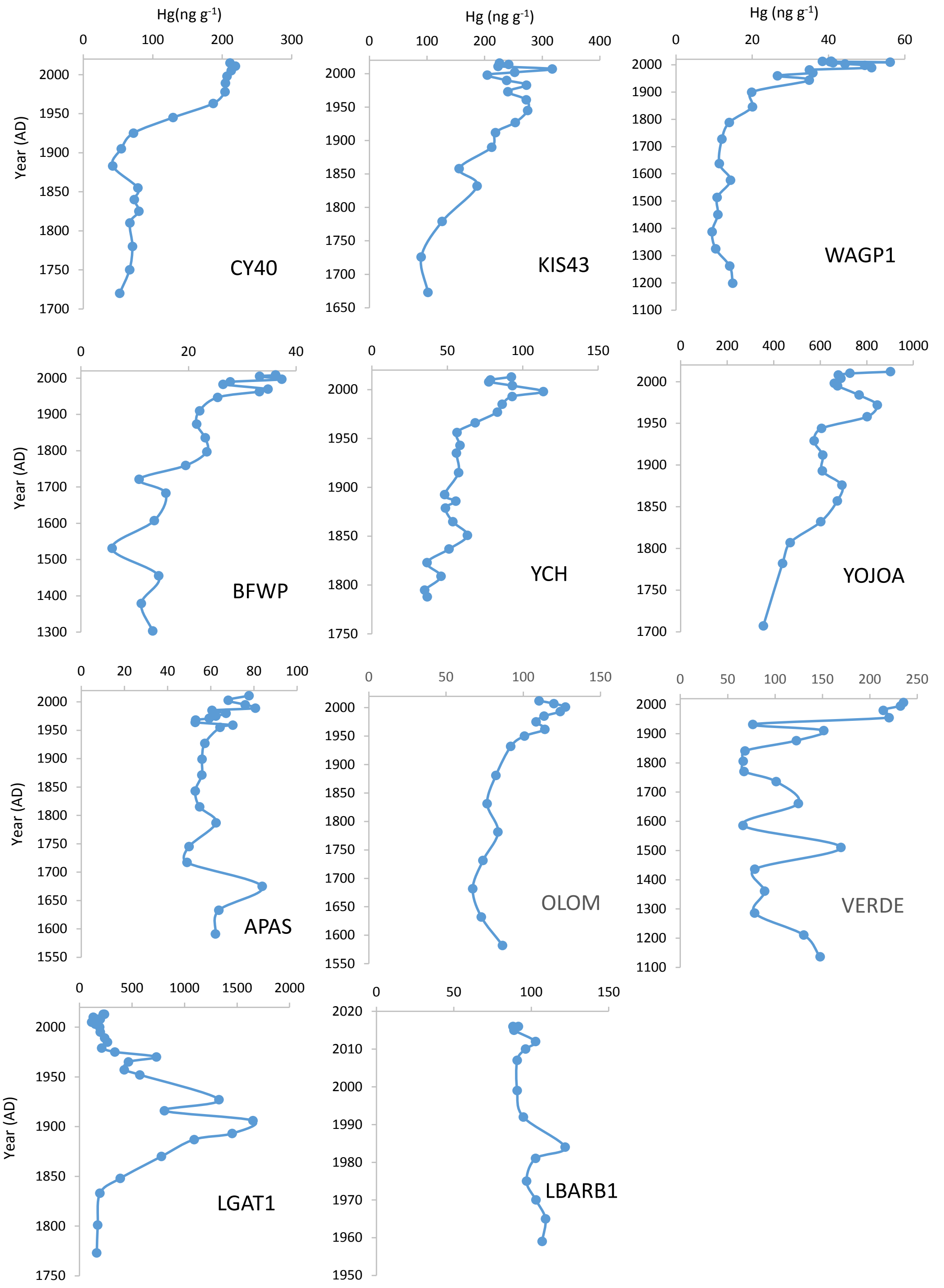

Figure S30. Mercury concentrations with time in the sediment cores. The dates out of ^{210}Pb chronologies were estimated by using the ^{210}Pb sedimentation rates (see the text of the article).

8. Mercury concentrations in the sediment cores

Table S2. Mercury concentrations in the sediment cores.

CY40		KIS43		YCH		WAGP1		BFWP	
Depth (cm)	Hg (ng g^{-1})	Depth (cm)	Hg (ng g^{-1})	Depth (cm)	Hg (ng g^{-1})	Depth (cm)	Hg (ng g^{-1})	Depth (cm)	Hg (ng g^{-1})
0-5	211.2	0-5	226.0	0-1	92.4	0-1	40.7	0-1	36.2
5-10	218.7	5-10	241.7	1-2	78.5	1-2	38.4	1-2	36.2
10-15	212.8	10-15	223.5	3-4	77.5	3-4	40.4	3-4	33.2
15-20	206.9	15-20	317.4	5-6	93.1	4-5	56.2	6-7	37.3
20-25	204.6	20-25	252.3	7-8	113.6	6-7	41.2	9-10	27.7
25-30	203.9	25-30	204.8	9-10	92.9	7-8	44.4	12-13	26.4
30-35	186.8	35-40	238.2	11-12	86.3	9-10	49.5	14-15	34.8
35-40	129.1	40-45	272.6	13-14	83.2	11-12	51.3	15-16	33.2
40-45	72.1	45-49	240.3	15-16	68.5	12-13	35.0	16-17	25.4
45-49	54.6	49-53	272.3	17-18	56.3	13-14	35.9	18-19	22.0
49-53	42.4	53-57	274.8	19-20	58.4	14-15	26.6	20-21	21.5
53-57	78.5	57-61	253.3	21-22	56.0	15-16	35.0	22-23	23.1
57-61	73.5	61-65	219.0	23-24	57.4	17-18	19.9	24-25	23.4
61-65	80.0	65-69	212.5	26-27	48.2	19-20	20.1	26-27	19.5
69-73	67.2	69-73	155.8	27-28	55.6	21-22	14.0	28-29	10.8
77-81	70.8	73-77	187.0	29-30	48.8	23-24	12.1	30-31	15.8
85-89	66.6	81-85	126.4	31-32	53.6	25-26	11.4	34-35	13.6
93-97	52.4	89-93	90.1	33-34	63.3	27-28	14.3	38-39	5.8
		97-101	101.6	35-36	51.1	29-30	10.8	42-43	14.5
				37-38	36.5	31-32	11.0	46-47	11.2
				39-40	45.7	33-34	9.5	50-51	13.3
				41-42	34.9	35-36	10.5		
				42-43	36.6	37-38	14.1		
						39-40	14.9		

Table S2. ***Continued.***

YOJOA		VERDE		APAS		OLOM		LGAT1		LBARB1	
Depth (cm)	Hg (ng g^{-1})	Depth (cm)	Hg (ng g^{-1})	Depth (cm)	Hg (ng g^{-1})	Depth (cm)	Hg (ng g^{-1})	Depth (cm)	Hg (ng g^{-1})	Depth (cm)	Hg (ng g^{-1})
0-1	901.6	0-1	235.4	0-0.5	77.7	0-1	110.2	0-1	226.8	0-1	91.7
1-2	727.4	1-2	232.2	1-1.5	68.0	3-4	119.9	3-4	237.3	3-4	88.2
2-3	677.5	2-3	214.2	2-2.5	75.9	6-7	127.4	6-7	131.5	6-7	88.8
4-5	688.8	3-4	220.2	3-3.5	80.6	9-10	123.9	9-10	197.2	9-10	102.7
6-7	660.6	4-5	76.4	4-4.5	60.6	12-13	113.5	13-14	115.9	12-13	96.3
8-9	673.8	5-6	151.2	5-5.5	67.0	15-16	108.3	15-16	150.1	15-16	90.9
10-11	766.8	6-7	122.3	6-6.5	62.3	18-19	114.1	18-19	192.2	18-19	90.9
12-13	844.7	7-8	68.2	7-7.5	59.2	21-22	100.8	21-22	198.2	20-21	94.9
14-15	801.3	8-9	66.2	8-8.5	53.0	24-25	91.9	24-25	238.9	24-25	121.8
16-17	605.6	9-10	67.1	9-9.5	52.8	27-28	82.3	27-28	265.0	28-29	102.8
18-19	573.4	10-11	101.1	10-10.5	70.2	30-31	76.6	30-31	211.3	30-31	97.0
20-21	610.8	12-13	124.5	11-11.5	64.3	33-34	83.6	33-34	335.5	32-33	103.1
22-23	609.1	14-15	66.1	12-12.5	57.2	36-37	73.9	36-37	732.8	36-37	109.2
24-25	693.4	16-17	169.4	14-14.5	55.9	39-40	67.2	39-40	464.8	38-39	107.0
26-27	673.3	18-19	78.6	16-16.5	55.8	42-43	72.8	42-43	425.4		
28-29	602.3	20-21	88.9	18-18.5	52.8	45-46	86.6	44-45	574.9		
30-31	471.0	22-23	78.5	20-20.5	54.8			48-49	1327.2		
32-33	437.9	24-25	130.2	22-22.5	62.4			51-52	808.1		
38-39	355.4	26-27	147.4	25-25.5	49.9			54-55	1650.9		
		29-30	104.4	27-27.5	49.0			57-58	1453.6		
				30-30.5	83.8			60-61	1091.8		
				33-33.5	63.7			65-66	780.0		
				36-36.5	62.1			70-71	388.2		
								75-76	194.5		
								79-80	172.2		
								85-86	163.6		

9. ^{210}Pb chronologies and sedimentation rates in the sediment cores.

Table S3. ^{210}Pb chronology of core CY40A taken from Lake Cypress, Florida.

Depth	Drymass	Chronology			Sedimentation Rate		
		Date	Age				
cm	g cm^{-2}	AD	yr	±	g cm^{-2} yr^{-1}	cm yr^{-1}	± %
0	0	2017	0				
2.5	0.1875	2015	2	2	0.0879	1.233	7.6
7.5	0.535	2011	6	2	0.0767	1.077	12.2
12.5	0.9	2005	12	2	0.0572	0.702	10.7
17.5	1.35	1998	19	2	0.0637	0.645	9
22.5	1.8875	1989	28	4	0.0582	0.515	12.5
27.5	2.48	1978	39	6	0.0515	0.401	12.9
32.5	3.1725	1963	54	6	0.0402	0.205	15.7
37.5	4.445	1945	72	6	0.0973	0.263	21.3
42.5	6.87	1925	92	9	0.146	0.238	27.6
47	10.1505	1905	112	12	0.1322	0.202	74.3
51	12.4205	1883	134	15	0.0657	0.16	56.3
55	13.4365	1855	162	23	0.0363	0.143	62.2

Table S4. ^{210}Pb chronology of core KIS43 taken from Lake Kissimmee, Florida.

Depth	Drymass	Chronology			Sedimentation Rate		
		Date	Age				
cm	g cm^{-2}	AD	yr	±	g cm^{-2} yr^{-1}	cm yr^{-1}	± %
0	0	2017	0				
2.5	0.0625	2016	1	2	0.0674	2.271	5.8
7.5	0.2225	2014	3	2	0.0573	1.56	8.9
12.5	0.43	2011	6	2	0.0902	1.992	11.5
17.5	0.675	2007	10	2	0.0507	0.97	6.4
22.5	0.9525	2002	15	2	0.0659	1.121	7.5
27.5	1.2625	1998	19	2	0.1012	1.557	8.3
32.5	1.6025	1995	22	2	0.0831	1.2	9
37.5	1.955	1990	27	2	0.0672	0.92	7.9
42.5	2.3325	1983	34	2	0.0407	0.524	10.8
47	2.6925	1973	44	2	0.0318	0.389	8.1
51	3.0285	1961	56	3	0.0266	0.305	10.9
55	3.3885	1945	72	4	0.018	0.188	17.7
59	3.7945	1927	90	6	0.0331	0.308	27.7
63	4.2485	1912	105	8	0.0255	0.211	33.3
67	4.7605	1890	127	12	0.0201	0.15	47.9
71	5.3245	1858	159	18	0.0151	0.11	59.9

Table S5. ^{210}Pb chronology of core YCH taken from Yaal Chac, Maxico.

Depth	Drymass	Chronology			Sedimentation Rate		
		Date	Age				
cm	g cm^{-2}	AD	yr	±	g cm^{-2} yr^{-1}	cm yr^{-1}	± %
0	0	2013	0				
0.5	0.0261	2013	0	2	0.0518	0.904	11.9
3.5	0.2005	2008	5	2	0.0307	0.481	9.6
6.5	0.4093	2001	12	2	0.0323	0.452	10.7
9.5	0.6287	1993	20	2	0.0201	0.291	9.1
12.5	0.8251	1982	31	2	0.0148	0.217	10.2
13.5	0.9028	1977	36	2	0.0175	0.166	13.4
15.5	1.1422	1966	47	3	0.0306	0.265	15.7
18.5	1.4797	1953	60	4	0.0227	0.187	17.1
21.5	1.8716	1935	78	6	0.0193	0.166	26
24.5	2.1768	1913	100	8	0.0094	0.098	42.7
25.5	2.2529	1907	106	8	0.0195	0.214	86.8
27.5	2.4511	1877	136	12	0.0024	0.025	41
28.5	2.5418	1823	190	13	0.0009	0.01	45.1

Table S6. ^{210}Pb chronology of core WAGP1 taken from Wallywash Great Pond, Jamaica.

Depth	Drymass	Chronology			Sedimentation Rate		
		Date	Age				
cm	g cm^{-2}	AD	yr	±	g cm^{-2} yr^{-1}	cm yr^{-1}	± %
0	0	2013	0				
0.5	0.0358	2013	0	2	0.1652	2.321	53.5
3.5	0.2492	2011	2	2	0.08	1.216	44.2
6.5	0.4304	2007	6	3	0.0324	0.424	31.3
9.5	0.7077	1998	15	5	0.0249	0.262	34.3
11.5	0.9056	1989	24	7	0.0211	0.186	42.5
12.5	1.0479	1981	32	10	0.0152	0.104	41.9
13.5	1.1974	1970	43	15	0.0113	0.072	61.6
14.5	1.3597	1959	54	21	0.0205	0.124	94.9
15.5	1.5275	1943	70	26	0.0056	0.029	114.8

Table S7. ^{210}Pb chronology of core BFWP taken from Freshwater Pond, Barbuda.

Depth	Drymass	Chronology			Sedimentation Rate		
		Date	Age				
cm	g cm^{-2}	AD	yr	±	g cm^{-2} yr^{-1}	cm yr^{-1}	± %
0	0	2010	0				
0.5	0.5	2009	1	2	0.3805	0.864	62.6
3.5	3.5	2005	5	2	0.2524	0.551	39
6.5	6.5	1997	13	4	0.1191	0.253	21.9
9.5	9.5	1990	20	5	0.4829	0.985	88.5
12.5	12.5	1983	27	6	0.1064	0.198	25.1
14.5	14.5	1970	40	10	0.0776	0.133	31.8
15.5	15.5	1963	47	15	0.0515	0.087	29.1
16.5	16.5	1947	63	22	0.034	0.057	65.8
18.5	18.5	1910	100	30	0.0326	0.054	95.1

Table S8. ^{210}Pb chronology of core YOJOA taken from Lake Yojoa, Honduras.

Depth	Drymass	Chronology			Sedimentation Rate		
		Date	Age				
cm	g cm^{-2}	AD	yr	±	g cm^{-2} yr^{-1}	cm yr^{-1}	± %
0	0	2013	0				
0.5	0.1365	2012	1	2	0.1369	0.583	8.9
2.5	0.587	2008	5	2	0.0852	0.411	7.3
4.5	0.9654	2004	9	2	0.0905	0.485	9.1
6.5	1.3334	1998	15	2	0.0526	0.345	8.2
8.5	1.5761	1995	18	2	0.1198	0.438	12.7
10.5	2.4282	1984	29	2	0.0482	0.143	10.7
12.5	2.921	1972	41	2	0.0395	0.165	9.2
14.5	3.387	1958	55	3	0.0271	0.132	12.9
16.5	3.7445	1944	69	4	0.022	0.128	15.7
18.5	4.0719	1929	84	5	0.0211	0.129	22.8
20.5	4.3958	1912	101	8	0.017	0.107	30.3
22.5	4.7106	1893	120	12	0.0161	0.102	49.9
24.5	5.0283	1876	137	16	0.019	0.116	60.8
26.5	5.3637	1857	156	19	0.0088	0.053	80.3

Table S9. ^{210}Pb chronology of core VERDE taken from Lagoon Verde, El Salvador.

Depth	Drymass	Chronology			Sedimentation Rate		
		Date	Age				
cm	g cm^{-2}	AD	yr	±	g cm^{-2} yr^{-1}	cm yr^{-1}	± %
0	0	2013	0				
0.5	0.1281	2007	6	2	0.0199	0.081	5.9
1.5	0.37	1995	18	2	0.021	0.087	7.2
2.5	0.612	1980	33	2	0.0116	0.048	7
3.5	0.8587	1955	58	3	0.0087	0.03	10.8
4.5	1.1966	1932	81	5	0.0331	0.096	30
5.5	1.544	1911	102	8	0.008	0.026	26.5
6.5	1.8244	1855	158	32	0.0025	0.008	33.3

Table S10. ^{210}Pb chronology of core APAS taken from Apastepeque Lagoon, El Salvador.

Depth	Drymass	Chronology			Sedimentation Rate		
		Date	Age				
cm	g cm^{-2}	AD	yr	±	g cm^{-2} yr^{-1}	cm yr^{-1}	± %
0	0	2013	0				
0.25	0.0861	2011	2	2	0.05	0.146	17.6
1.25	0.4267	2003	10	2	0.0326	0.097	17.3
2.25	0.7565	1995	18	3	0.0526	0.162	24.2
3.25	1.0746	1989	24	4	0.0581	0.198	25.8
5.25	1.6385	1980	33	5	0.0617	0.221	32.3
6.25	1.913	1975	38	6	0.0741	0.228	51
8.25	2.612	1968	45	7	0.1008	0.272	65.4
9.25	3.0243	1964	49	8	0.0533	0.121	50.1
10.25	3.4905	1959	54	8	0.0974	0.21	56.4
11.25	3.9501	1955	58	9	0.0329	0.073	45
12.25	4.3898	1941	72	12	0.0322	0.073	65.8
13.25	4.8266	1919	94	20	0.0119	0.028	68.4

Table S11. ^{210}Pb chronology of core OLOM taken from Lagoon Olomega, El Salvador.

Depth	Drymass	Chronology			Sedimentation Rate		
		Date	Age				
cm	g cm^{-2}	AD	yr	±	g cm^{-2} yr^{-1}	cm yr^{-1}	± %
0	0	2013	0				
0.5	0.1072	2012	1	2	0.124	0.601	39.5
2.5	0.5162	2009	4	2	0.1179	0.566	30.1
4.5	0.9398	2005	8	2	0.1299	0.626	38.7
6.5	1.3467	2001	12	2	0.0811	0.411	23.7
8.5	1.7297	1996	17	3	0.06	0.316	28
10.5	2.1056	1990	23	4	0.0674	0.365	27.7
12.5	2.4688	1985	28	4	0.0678	0.362	41.5
14.5	2.8544	1979	34	5	0.0815	0.422	45.3
16.5	3.2415	1972	41	6	0.0312	0.158	33.9
18.5	3.6439	1962	51	8	0.0662	0.327	75.1
20.5	4.0504	1955	58	10	0.0433	0.211	68.3
22.5	4.4645	1945	68	12	0.0387	0.176	68
24.5	4.93	1932	81	15	0.0358	0.148	78.9
26.5	5.4286	1914	99	24	0.0125	0.047	85.2

Table S12. ^{210}Pb chronology of core LGAT1 taken from Gatun Lake, Panama.

Depth	Drymass	Chronology			Sedimentation Rate		
		Date	Age				
cm	g cm^{-2}	AD	yr	±	g cm^{-2} yr^{-1}	cm yr^{-1}	± %
0	0	2013	0				
0.5	0.0056	2013	0	2	0.2565	1.608	48.3
3.5	0.0373	2013	0	2	0.7308	4.098	
6.5	0.1154	2010	3	2	0.1339	0.834	31
9.5	0.1714	2008	5	2	0.3628	2.328	83
15.5	0.3461	2003	10	3	0.1392	0.835	33
18.5	0.4328	2000	13	4	0.1195	0.74	38
20.5	0.5514	1996	17	4	0.0745	0.474	25.4
24.5	0.6636	1989	24	5	0.0935	0.59	45.2
29.5	0.7788	1981	32	7	0.1262	0.751	70.8
34.5	0.9076	1973	40	8	0.0954	0.543	68
39.5	1.0536	1965	48	10	0.1328	0.754	87.6
44.5	1.2074	1952	61	14	0.0368	0.2	61.3
49.5	1.5475	1923	90	28	0.028	0.15	107.9

Table S13. ^{210}Pb chronology of core LBARB1 taken from Lake Barbacoas, Colombia.

Depth	Drymass	Chronology			Sedimentation Rate		
		Date	Age				
cm	g cm^{-2}	AD	yr	±	g cm^{-2} yr^{-1}	cm yr^{-1}	± %
0	0	2016	0				
0.5	0.1155	2016	0	2			
3.5	1.4348	2016	0	2			
6.5	2.7814	2015	1	2	1.3937	2.991	89.7
9.5	4.2308	2012	4	3	0.2434	0.531	26.8
12.5	5.5345	2010	6	3	0.6951	1.677	677.4
15.5	6.717	2007	9	3	0.196	0.52	30.4
18.5	7.797	1999	17	4	0.0935	0.298	25
20.5	8.2869	1992	24	4	0.0476	0.177	24.7
22.5	8.8713	1986	30	5	0.3933	1.438	96.7
24.5	9.3807	1984	32	5	0.1377	0.604	44.5
26.5	9.7834	1983	33	5	0.3782	1.964	54.7
28.5	10.1511	1981	35	5	0.0564	0.314	28.4
30.5	10.5029	1975	41	5	0.0526	0.301	43.5
32.5	10.8499	1970	46	5	0.1096	0.646	85.6
34.5	11.182	1966	50	5	0.0747	0.458	64.8
36.5	11.5026	1965	51	6	0.308	1.735	79
38.5	11.8921	1959	57	6	0.0821	0.431	76

10. Mercury fluxes in the sediment cores

Table S14. Mercury fluxes (μg m^{-2} yr^{-1}) in the sediment cores.

CY40		KIS43		YCH		WAGP1		BFWP	
Date	Hg fluxes	Date	Hg fluxes	Date	Hg fluxes	Date	Hg fluxes	Date	Hg fluxes
2015	185.6	2016	152.3	2013	47.9	2013	67.2	2009	137.6
2011	167.8	2014	138.5	2010	31.4	2012	46.1	2008	123.0
2005	121.7	2011	201.6	2008	23.8	2011	32.3	2005	83.8
1998	131.8	2007	160.9	2004	29.8	2009	28.1	1997	44.5
1989	119.1	2002	166.3	1998	28.4	2007	13.4	1990	78.5
1978	105.0	1998	207.3	1993	18.7	2003	13.3	1983	28.1
1963	75.1	1990	160.1	1985	12.8	1998	12.3	1970	27.0
1945	125.6	1983	110.9	1977	14.6	1989	10.8	1963	17.1
1925	102.8	1973	76.4	1966	21.0	1981	5.3	1947	8.6
1905	72.1	1961	72.4	1956	13.0	1970	4.1	1910	7.2
1883	27.9	1945	49.5	1943	12.8	1959	5.5	1874	7.1
1855	28.5	1927	83.8	1935	10.8	1943	3.5	1836	7.6
1840	39.8	1912	55.8	1915	6.9	1899	2.4		
		1890	42.7	1892	6.6	1845	2.5		
		1858	31.2	1886	7.6				
		1832	37.4	1879	6.6				
				1865	7.3				
				1850	8.6				

Table S14. ***Continued.***

YOJOA		VERDE		APAS		OLOM		LGAT1		LBARB1	
Date	Hg fluxes	Date	Hg fluxes	Date	Hg fluxes	Date	Hg fluxes	Date	Hg fluxes	Date	Hg fluxes
2012	1234.2	2007	46.8	2011	38.8	2012	136.7	2013	581.6	2016	1283.2
2010	800.1	1995	48.8	2003	22.2	2007	143.9	2013	1734.0	2016	1234.7
2008	577.2	1980	24.8	1995	39.9	2001	103.3	2010	176.1	2015	1237.5
2004	623.4	1955	19.2	1989	46.8	1993	74.4	2008	715.5	2012	250.0
1998	347.5	1932	25.3	1985	36.3	1985	76.9	2005	231.8	2010	669.2
1995	807.2	1911	12.1	1980	41.4	1975	54.2	2003	208.9	2007	178.2
1984	369.6	1876	9.8	1975	46.2	1962	75.5	2000	229.7	1999	85.0
1972	333.7	1841	5.5	1971	47.4	1950	42.3	1995	158.5	1992	45.2
1958	217.1			1968	53.4	1932	32.9	1989	223.4	1984	167.7
1944	133.2			1964	28.1	1881	24.7	1985	249.1	1981	58.0
1929	121.0			1959	42.1	1831	23.0	1979	266.2	1975	51.0
1912	103.8			1955	21.2			1975	318.7	1970	113.0
1893	98.1			1927	18.4			1970	732.8	1965	336.3
1876	131.7			1899	17.9			1965	617.3	1959	87.8
1857	87.5			1871	17.9			1957	425.4		
1832	78.3			1843	16.9			1952	212.7		
1807	61.2							1927	398.2		
1782	56.9							1916	439.6		
1707	46.2							1906	898.1		
								1893	790.8		
								1887	593.9		
								1870	424.3		
								1848	211.2		
								1833	105.8		
								1801	93.7		

11. Mercury flux ratios (against 1850 values, except the cores from lakes Yojoa, Gatun and Barbacoas) versus time in the sediment cores from the study sites

Table S15. Mercury flux ratios against the 1850 fluxes, except Yojoa (the 1700 flux), Gatun (the 1800 flux), as local human activities occurred in both lakes around the 1850s, and Barbacoas (the 1990 flux, the lowest in the core), as the core covers the 1960s – 2016.

CY40		KIS43		YCH		WAGP1		BFWP	
Year	Flux ratio	Year	Flux ratio	Year	Flux ratio	Year	Flux ratio	Year	Flux ratio
2015	6.6	2016	4.9	2013	6.0	2013	27.3	2009	19.1
2011	6.0	2014	4.5	2010	3.9	2012	18.8	2008	17.1
2005	4.3	2011	6.5	2008	3.0	2011	13.2	2005	11.6
1998	4.7	2007	5.2	2004	3.7	2009	11.5	1997	6.2
1989	4.3	2002	5.4	1998	3.6	2007	5.5	1990	10.8
1978	3.8	1998	6.7	1993	2.3	2003	5.4	1983	3.9
1963	2.7	1990	5.2	1985	1.6	1998	5.0	1970	3.7
1945	4.5	1983	3.6	1977	1.8	1989	4.4	1963	2.4
1925	3.7	1973	2.5	1966	2.6	1981	2.2	1947	1.2
1905	2.6	1961	2.3	1956	1.6	1970	1.7	1910	0.99
1883	1.0	1945	1.6	1943	1.6	1959	2.2	1874	0.98
1855	1.0	1927	2.7	1935	1.3	1943	1.4	1858	1.0
1850	1	1912	1.8	1915	0.86	1899	1.0	1850	1
		1890	1.4	1892	0.82	1850	1		
		1858	1.0	1886	0.94				
		1850	1	1879	0.83				
				1865	0.91				
				1850	1				

Table S15. ***Continued.***

YOJOA		VERDE		APAS		OLOM		LGAT1		LBARB1	
Year	Flux ratio	Year	Flux ratio	Year	Flux ratio	Year	Flux ratio	Year	Flux ratio	Year	Flux ratio
2012	26.8	2007	8.6	2011	2.3	2012	6.2	2013	5.8	2016	28.5
2010	17.4	1995	8.9	2003	1.3	2007	6.5	2013	17.3	2016	27.4
2008	12.5	1980	4.5	1995	2.4	2001	4.7	2010	1.8	2015	27.5
2004	13.6	1955	3.5	1989	2.8	1993	3.4	2008	7.2	2012	5.6
1998	7.6	1932	4.6	1985	2.2	1985	3.5	2005	2.3	2010	14.9
1995	17.5	1911	2.2	1980	2.4	1975	2.5	2003	2.1	2007	4.0
1984	8.0	1876	1.8	1975	2.7	1962	3.4	2000	2.3	1999	1.9
1972	7.3	1850	1	1971	2.8	1950	1.9	1995	1.6	1992	1.0
1958	4.7			1968	3.2	1932	1.5	1989	2.3	1984	3.7
1944	2.9			1964	1.7	1881	1.1	1985	2.5	1981	1.3
1929	2.6			1959	2.4	1850	1	1979	2.7	1975	1.1
1912	2.3			1955	1.3			1975	3.2	1970	2.5
1893	2.1			1927	1.1			1970	7.3	1965	7.5
1876	2.9			1899	1.1			1965	6.2	1959	1.9
1857	1.9			1871	1.1			1957	4.3		
1700	1			1850	1			1952	2.1		
								1927	4.0		
								1916	4.4		
								1906	9.0		
								1893	7.9		
								1887	5.9		
								1870	4.2		
								1850	2.1		
								1800	1		

12. How increased anthropogenic Hg emissions could affect Hg fluxes into the lake sediments (Table S16).

Streets et al. published global and regional trends of Hg emission for 2010 - 2015 in 2019 (see below, from Streets et al., 2019, Atmospheric Environment 201, 417-427). In central America, Hg emissions increase slowly with a maximum increase rate (5.42%) from 2010 to 2015. However, this increase follows a decrease from 2000, and so the 2000 and 2015 values show similar Hg emission levels, which does not match the observed sediment Hg increase. Even if we assume that Hg emissions in central America keep increasing at the same rate from 2001 to 2015, by 2015, Hg emission is only doubled.

Here we estimate how this doubled Hg emission could affect Hg fluxes in the lake sediments. UN Environment 2019 suggested anthropogenic Hg emission makes 30% contribution to the Hg in the atmosphere (UN Environment 2019). We assume that anthropogenic Hg contribution to the atmosphere is 30% in 2001, and contributions from environmental cycling and geology sources to the atmosphere remain the same since 2001, then the doubled anthropogenic Hg emission could make a 30% Hg increase in the

atmosphere in 2015 in the region, which means atmospheric Hg deposition could have a 30% increase, or the increase in anthropogenic Hg emission makes an increase of 30% in the 2015 sediment Hg flux compared to the 2001 value. In the same way, we assume anthropogenic Hg contribution to the atmosphere is 30% in 2015. As we also assume that anthropogenic Hg emission is doubled from 2001 to 2015, then half of the anthropogenic Hg contribution to the atmosphere is from the increased emission, and this work out that the doubled anthropogenic Hg emission only makes an increase of 17.6% in the 2015 sediment Hg flux compared to the 2001 value. By contrast, we have observed a 3-fold increase since around 2000 in the remote lake sediment Hg fluxes. Furthermore, the actual 2000 and 2015 Hg emission data from the Table S16 (below) for central America would suggest little contribution to the sediment Hg increase while in the broader American region, Hg emissions show little net increase from 2000 to 2015. Therefore, we believe that it is reasonable to say the changes in Hg emission and deposition to remote lakes do not match the increase in sediment Hg fluxes in recent years and therefore that anthropogenic Hg emission is not a primary driver for the Hg increase in the sediments, at least in these remote lakes.

Table S16. Annual emissions of mercury by region (unit:Mg/yr) (Streets et al., 2019).

region	2000	2010	2011	2012	2013	2014	2015	2010-2015 growth (%/yr)
Canada	12.7	9.8	9.1	8.7	8.6	8.7	8.3	-3.16
USA	127.7	73.1	66.9	59.1	55.5	50.6	42.7	-10.19
Central America	33.5	26.4	29.6	31.1	32.1	33.1	34.3	5.42
South America	239.0	266.0	279.6	279.6	270.9	271.5	275.8	0.73
Sub-total	412.9	375.3	385.2	378.5	367.1	363.9	361.1	
Global total	1964	2188	2300	2330	2337	2360	2389	1.78

13. References for SI:

Appleby, P. G., 2001. Chronostratigraphic techniques in recent sediments. In W M Last and J P Smol (eds.) Tracking Environmental Change Using Lake Sediments. Vol. 1: Basin Analysis, Coring, and Chronological Techniques. Kluwer Academic Publishers, Dordrecht. Pp171-203.

Drevnick, P.E., Cooke, C.A., Barraza, D., Blais, J.M., Coale, K.H., 2016. Spatiotemporal patterns of mercury accumulation in lake sediments of western North America. Science of Total Environment. 568: 1157-1170.

Streets, D. G., Horowitz, H. M., Lu, Z., Levin, L., Sunderland, E. M., 2019, Global and regional trends in mercury emissions and concentrations, 2010-2015. Atmospheric Environment 201, 417-427

Swain, E. B., Engstrom, D. R., Brigham, M. E., Henning, T. A., Brezonik, P. L., 1992. Increase rates of atmospheric mercury deposition in Midcontinential North America. Science. 257: 784-787.

Yang, H., 2015. Lake Sediments May Not Faithfully Record Decline of Atmospheric Pollutant Deposition. Environ. Sci. Technol. 49: 12607-12608.
